# New Temporin A Analogues Modified in Positions 1 and 10—Synthesis and Biological Studies

**DOI:** 10.3390/pharmaceutics17040396

**Published:** 2025-03-21

**Authors:** Dilyana Dimitrova, Veronica Nemska, Ivan Iliev, Stoyko Petrin, Nelly Georgieva, Dancho Danalev

**Affiliations:** 1 Biotechnology Department, University of Chemical Technology and Metallurgy, 8 Kliment Ohridski Blvd., 1797 Sofia, Bulgaria; dilyana@uctm.edu (D.D.); vnemska@uctm.edu (V.N.); stpetrin@uctm.edu (S.P.); neli@uctm.edu (N.G.); 2 Institute of Experimental Morphology, Pathology and Anthropology with Museum, Bulgarian Academy of Sciences, Acad. G. Bonchev Str., bl. 25, 1113 Sofia, Bulgaria; taparsky@abv.bg

**Keywords:** antimicrobial peptides, Temporin A, antimicrobial activity, antiproliferative activity, cytotoxicity, phototoxicity, fluorinated amino acid, hydrolytic stability

## Abstract

**Background/Objectives**: With growing antimicrobial resistance, the overuse of antibiotics, and stagnation in the discovery of new antibiotics, a novel alternative is required to overcome hard-to-treat infections. Antimicrobial peptides (AMPs) show great potential as a possible alternative to standard chemotherapeutics. Temporins are a group of AMPs that have been under the spotlight in numerous studies. Herein, we report the design and synthesis of Temporin A modified in position 1, where the proteinogenic amino acid Phe is replaced by Tyr or fluorinated Phe. In addition, in other analogues, in position 10, the Ser residue is replaced by Tyr or Thr. The aim of all modifications in the primary structure of the native Temporin A is to study the influence of the changes made on the antibacterial properties, antiproliferative activity, and hydrolytic stability of the newly synthesized molecules. **Methods**: The Fmoc/OBu^t^ SPPS strategy was employed for the synthesis of the novel-designed analogues. The antibacterial activity was evaluated with both disk diffusion and broth microdilution methods. The BALB 3T3 NRU test and MTT dye reduction assay were used to determine safety and antiproliferative activity. **Results**: The investigated analogues have low toxicity and are photosafe. The greatest selectivity was shown by DTTyr10 towards MCF-7 cells. DT4F, containing fluorinated Phe in position 1, was the most effective antibacterial agent among the new compounds. The incorporation of Thr in position 10, in comparison with the natural Ser residue, led to an increase in the antiproliferative effect of the new peptide. **Conclusions**: The obtained structure–activity relationship data show that the most promising compound in the tested series is FLPLIGRVL-**Y**-GILNH_2_, where the Ser residue in position 10 is replaced by a more hydrophobic OH-containing Tyr residue. The analogue containing fluorinated Phe in position 1, DT4F, has the highest antiproliferative effect against both tested tumor cell lines, combined with good antibacterial properties at the lowest MIC (80 µg/mL), but it is more cyto- and phototoxic than the parent DTA molecule and is not stable at pH 9 for a 24 h period.

## 1. Introduction

The capacity of microorganisms to endure and remain viable when exposed to antimicrobial agents is known as antimicrobial resistance (AMR), which currently represents one of the biggest concerns for public health. The infections caused by antimicrobial-resistant organisms are not only hard to cure, but they also carry a constant risk of serious illness and even death [1]. The main way microorganisms become resistant to antibiotics is their widespread use and abuse. Searching for novel antibiotics is one of the policies that the World Health Organization recommends. However, since the discovery of linezolid and daptomycin in the 1980s, the only newly marketed drugs have been combinations or optimizations of previously identified compounds [2]. Thus, many scientific groups turn their attention to other natural molecules with antimicrobial activity. Among these substances, antimicrobial peptides (AMPs) have been recognized. They are a promising class of compounds due to their variety of killing mechanisms against bacterial infections. The in vitro testing of both the minimal inhibitory concentrations (MICs) and minimal bactericidal concentrations (MBCs) of AMPs reveals their ability to eradicate multidrug-resistant bacteria [3]. Most living organisms express AMPs, which are crucial for adaptive immunity throughout the onset of autoimmune disorders and cancers, as well as for defense against bacterial, viral, and fungal infections. AMPs have the potential to be effective against infections caused by bacteria as well as other pathogens that are resistant to the current antibiotics. The two main mechanisms by which AMPs eliminate pathogenic infections are as follows:

- Direct killing by means of damage to the integrity of the membrane;
- Interference with the production of intracellular components, such as proteins and nucleic acids.

AMPs also exhibit a variety of other actions, including membrane destabilization and depolarization [3]. Last but not least, peptides possess a number of valuable properties, such as small size [4] and easy penetration through cell membranes [5]. In addition, the development of modern methods for peptide synthesis allows for their easy synthesis in pure form [6].

According to some experimental data, the total negative charge of the tumor surface and the bacterial membrane due to the presence of anionic molecules like heparin sulfate and glycoconjugates theoretically explains why many AMPs also have anticancer activity [7,8,9,10].

Among all AMPs, temporins are one of the most researched groups. Originally, they were isolated from the skin of the European red frog (*Rana temporaria*). They are amidated at the C-terminus due to a post-translational enzymatic reaction, have a low net charge (0 to +3) in neutral pH, and are 8–14 amino acids in length. The main positive feature of temporins is that they have no effect on eukaryotic cells and are especially effective against Gram-positive bacteria. Because of the cationic nature of the latter, temporins cause cell lysis by specifically targeting the cytoplasmic membranes of microorganisms [11].

In order to investigate the impact of modification of the Temporin A molecule (F^1^L^2^P^3^L^4^I^5^G^6^R^7^V^8^L^9^S^10^G^11^I^12^L^13^-NH_2_) on its antibacterial activity, hydrolytic stability, and antiproliferative activity, the following substitutions were made:

- Phe^1^ was replaced with another proteinogenic aromatic amino acid Tyr or non-proteinogenic fluorinated phenylalanine (Phe(4-F));
- Ser^10^ was substituted with other hydroxyl-containing natural amino acids (Tyr and Thr).

All changes were designed to mimic the naturally present amino acids in the primary structure of Temporin A with similar natural or non-proteinogenic amino acids, in order to study their importance for biological activity and stability. Taking into account similar mechanisms of action of antimicrobial and anticancer peptides, the phototoxicity, cytotoxicity, and antiproliferative ability of the novel peptides were also examined and compared to those of the parent peptide Temporin A.

## 2. Materials and Methods

### 2.1. Synthesis and Analytical Data

The specifically protected amino acids needed for the synthesis of the targeted peptides Fmoc-L-Leu-OH, Fmoc-L-Phe-OH, Fmoc-L-Phe(4-F)-OH, Fmoc-L-Val-OH, Fmoc-L-Pro-OH, Fmoc-L-Tyr(tBu)-OH, Fmoc-Gly-OH, Fmoc-L-Arg(Pbf)-OH, Fmoc-L-Ile-OH, Fmoc-L-Thr(OtBu)-OH, Fmoc-L-Lys(Boc)-OH, and Fmoc-L-Ser(Trt)-OH, as well as the polymer carrier Fmoc-Rink-Amid-MBHA Resin, and activation agents N,N,N′,N′-Tetramethyl-O-(1H-benzotriazol-1-yl)uronium hexafluorophosphate (HBTU), N,N′-Diisopropylcarbodiimide (DIC), 2-(1H-Benzotriazole-1-yl)-1,1,3,3-tetramethylaminium tetrafluoroborate (TBTU), or Benzotria-zol-1-yloxy)tripyrrolidinophosphonium hexafluorophosphate (PyBOP), base N,N-diisopropylethylamine (DIPEA), scavenger triisopropylsilane (TIS), and trifluoroacetic acid (TFA) were acquired from Iris Biotech GmbH (Marktredwitz, Germany). The additive N-Hydroxysuccinimide (1-HOSu) was purchased from Fluka Chemie AG (Darmstadt, Germany). The solvents for the synthesis dichloromethane (DCM) and N,N’-dimethylformamide (DMF) are from Valerus Ltd. (Sofia, Bulgaria). All reagents and solvents were used without pretreatment.

Targeted compounds were synthesized by means of solid-phase peptide synthesis with the Fmoc(9-fluorenylmethyloxycarbonyl)/O*t*-Bu strategy on a Rink-amide MBHA resin. HBTU, DIC, PyBOP, or TBTU were used for the activation of targeted amino acids in the following equivalent ratios: amino acid/HBTU(or PyBOP or TBTU)/DIPEA/resin—3/3/3/9/1; amino acid/DIC/1-HOSu/resin—3/3/3/1. Fmoc-group deprotection was performed using 20% piperidine/DMF. Both standard Kaiser and Chloranil tests were applied for the monitoring of the coupling and deprotection reactions. The cleavage of the final peptides from the resin as well as the deprotection of the protecting groups of the used amino acids was carried out using a cocktail of 95% trifluoroacetic acid (TFA), 2.5% triisopropylsilane (TIS), and 2.5% distilled water. Afterwards, every peptide was extracted as a filtrate in TFA and was precipitated using dry, cold diethyl ether. Furthermore, the precipitate was filtered and HPLC-MS/MS analyses were performed on an RP-HPLC Agilent Poroshell 120, 100 mm × 4.6 mm column in the Shimadzu LC MS/MS 8045 system with a flow rate of 0.30 mL/min, a column temperature of 40 °C, and a linear binary gradient, consisting of mobile phase A: H_2_O (10% Acetonitrile (AcCN); 0.1% HCOOH) and mobile phase B: AcCN (5% H_2_O; 0.1% HCOOH). The created gradient was applied as follows: 0.01 min → 10 min—80% A: 20% B → 5% A: 95% B; 10 min → 15 min—5% A: 95% B → 5% A: 95% B; 15 → 15.5 min—5% A: 95% B → 80% A: 20% B; 15.5 min → 22 min—80% A: 20% B → 80% A: 20% B.

The ESI(+)-MS in SCAN mode was used for proving the structures of targeted peptides. The system parameters were as follows: nebulizing gas flow: 3 L/min; heating gas flow: 10 L/min; interface temperature: 350 °C; DL temperature: 200 °C; heat block temperature: 400 °C; and drying gas flow: 10 L/min.

Optical rotation for all peptides was measured on an automated standard polarimeter PolamatA (Carl Zeis, Jena; Anton Paar Opto Tec GmbH, Seelze, Germany) in methanol solution at c = 1. A semi-automatic melting pointmeter (M3000, A. KRÜSS Optronic GmbH, Hamburg, Germany) was used for the measurement of the melting points. All analytical data are summarized in Table 1.

### 2.2. Safety Testing and Antiproliferative Potential

Mouse embryonic fibroblasts (BALB 3T3 clone A31), human breast epithelial cells (MCF-12F), luminal A breast cancer (MCF-7), and basal B-type breast cancer (MDA-MB-231) were used in in vitro experiments. Cell cultures were purchased from the American Type Cultures Collection (ATCC, Manassas, VA, USA). Cells were cultured in a growth medium (DMEM-4.5 g/L glucose), 10% FBS, and antibiotics (Sigma-Aldrich, Schnelldorf, Germany). Cells were incubated at 5% CO_2_, 37 °C, and 95% humidity. Plastic flasks (75 cm^2^) were used for the cell culture (Biologix, Lenexa, KS, USA).

#### 2.2.1. Safety Testing

The safety testing was performed according to the OECD Guidelines for the Testing of Chemicals, Section 4, Test No. 432. Mouse embryonic fibroblasts (BALB 3T3, clone A31) were used to perform the in vitro cytotoxicity and phototoxicity tests. Cells were plated at 1 × 10^4^ cells per well in 96-well cell culture plates. The cells were incubated in a thermostat for 24 h before the test peptide analogues were added. The stock solution of peptide analogues in DMSO had a concentration of 200 mM. Working concentrations (4–1000 µM) of peptides were obtained by diluting the stock solution with a culture medium. The phototoxicity test was performed using 96-well microplates. The microplates were irradiated with a dose of 2.4 J/cm^2^ using a solar light simulator Helios-iO (SERIC Ltd., Tokyo, Japan). After treatment with a neutral red (NR) medium (NR dissolved in culture medium, 34 µg/mL), washing, and application of the NR Desorb solution (freshly prepared 49 parts water + 50 parts ethanol + 1 part acetic acid), the absorption was measured on a microplate reader at a wavelength of 540 nm. Cytotoxicity was expressed as CC_50_ values.

#### 2.2.2. Antiproliferative Activity

The antiproliferative activity of the peptide analogues was determined by an MTT assay [13]. Normal epithelial cells (MCF-12F) and tumor cell lines (MCF-7 and MDA-MB-231) were used in the in vitro experiments. Cells were plated at 1 × 10^3^ cells/100 µL/well in 96-well microplates. After 24 h of incubation in a thermostat under standard conditions, the cells were treated with test compounds for 72 h. The optical density of the formazan was measured at λ = 540 nm using a microplate reader. Antiproliferative activities were expressed as IC_50_ values. The statistical analysis of the results was performed using one-way ANOVA, followed by Bonferroni’s post hoc test.

### 2.3. Antimicrobial Assay

In order to evaluate the antimicrobial activity, the parent peptide Temporin A and newly synthesized analogues were tested against model strains *Escherichia coli* NBIMCC 8785, *Pseudomonas aeruginosa* NBIMCC 3700, *Bacillus subtilis* NBIMCC 3562, *Arthrobacter oxydans* NBIMCC 9333, and *Candida albicans* NBIMCC 74. The strains were purchased from the National Bank for Industrial Microorganisms and Cell Cultures (NBIMCC, Sofia, Bulgaria). They were cultured as follows:

- *E. coli* 8785 in Luria–Bertani (LB, HiMedia, Mumbai, India) agar medium;
- *B. subtilis* 3562 in nutrient broth (NB, HiMedia, Mumbai, India) agar medium;
- *P. aeruginosa* 3700 and *A. oxydans* 9333 in Meat Peptone agar (MPA) medium;
- *C. albicans* 74 in Yeast Mold (YM) agar medium.

The cultivation was performed in an incubator shaker ES-20/60 (Biosan, Riga, Latvia), set for *B. subtilis* 3562, *A. oxydans* 9333, and *C. albicans* 74 at 30 °C, and for *E. coli* 8785 and *P. aeruginosa* 3700 at 37 °C, for 24 h with 120 rpm. The microbial cultures were then brought to a 0.5 McFarland turbidity using a Grant-bio DEN-1B densitometer (Grant Instruments, Nether Alderley, United Kingdom).

#### 2.3.1. Disk Diffusion Method

The disk diffusion method was employed as an additional technique in order to determine the percentage of inhibition of the synthesized peptides compared to a chosen antibiotic, used as a positive control. The antibiotics used were gentamicin (10 µg/disk), chloramphenicol (30 µg/disk), and itraconazole (10 µg/disk). In total, 100 μL from each microbial culture was spread on LB/NB/MPA/YM agar plates. The plates were then incubated at the appropriate temperature for a period of 30 min intended for the penetration of the culture into the agar. Afterwards, 20 µL of the peptide solutions with concentrations of 1.4 mg/mL or 10 mg/mL was pipetted on 6 mm sterile paper disks (HiMedia, Mumbai, India), and the disks were put on the agar surface. As a negative control, 10% EtOH/H_2_O was used. An antibiotic disk, corresponding to the strain (HiMedia, Mumbai, India), was also used as a positive control. Further, the agar plates were incubated at the desired temperature for 24 h. The antimicrobial activity was assessed by measuring the diameter of the inhibition zones in mm. The experiments were performed in triplicate and the average values of the triplicates were estimated. In addition, the percentage of inhibition was calculated as the correlation between the inhibition zone of the peptide (measured in mm) over the inhibition zone of the antibiotic (measured in mm) multiplied by 100.

#### 2.3.2. Determination of Minimal Inhibitory Concentration (MIC)

The broth dilution method was employed to estimate the MIC of the peptides. In total, 10 mg/mL peptide stock solutions in 10% EtOH/H_2_O were prepared. Microbial culture in the corresponding media and peptide solutions in a concentration range from 0 to 320 µg/mL (concentration levels: 0, 10, 20, 40, 80, 160, and 320 µg/mL) were placed in 96-well U-shaped-bottom polystyrene plates (Deltalab S.L., Barcelona, Spain). In addition, 10% EtOH/H_2_O control, diluted in the nutrient medium, was added in in separate wells. Furthermore, the microplates were incubated under aerobic conditions at the desired temperature for 24 h. The MIC (µg/mL) was considered to be the antimicrobial concentration at which the strain was clearly inhibited. The absorbance was measured at 630 nm (microplate reader PKL PPC 142, Italy). In total, 10 μL solution with concentrations greater than or equal to the MIC value was placed on the corresponding solid nutrient medium in order to evaluate a potential minimum bactericidal concentration (MBC) and minimal fungicidal concentration (MFC). Furthermore, the Petri dishes were incubated for 24 h at either 30 or 37 °C. Every experiment was carried out three times.

### 2.4. Hydrolytic Stability

The conditions in the stomach, blood plasma, and small intestine of the human organism were mimicked by three model pH systems in order to study the hydrolytic stability of the recently synthesized compounds. The model solutions were made in accordance with the *European Pharmacopoeia*, tenth edition, and they were additionally modified including particular enzymes able to hydrolyze peptides:

1. System for pH 2.0:
  - Solution A: In a volumetric flask, 119.0 mL of 0.1 mol/L HCl and 6.57 g of KCl in CO_2_-free water were combined, and the final solution was finished with 1000.0 mL of distilled water.
  - Solution B: A volumetric flask containing 5 mg of pepsin was filled to the brim with solution A. Consequently, the final model solution had a pH of 2.0 and a pepsin concentration of 0.5 mg/mL.
2. System for pH 7.4: To achieve a final trypsin concentration of 0.1 mg/mL in a model system with pH 7.4, amounts of 0.1 g trypsin, 2.38 g Na_2_HPO_4_, 0.19 g KH_2_PO_4_, and 8.0 g NaCl were dissolved to a total of 1000.0 mL in distilled water. One milliliter of blood plasma (ACCUCLOTM Reference plasma, Normal, Sigma Diagnostics) was recovered using fifteen milliliters of the pH 7.4 buffer that was obtained.
3. System for pH 9.0: In total, 420.0 mL of a solution containing 0.1 mol/L NaOH in distilled water was combined with 1000 mL of a solution containing 6.18 g H_3_BO_3_ in 0.1 mol/L KCl in distilled water. To reach the final concentration of 0.1 mg/mL of trypsin, an additional 0.1 mg of trypsin was dissolved in the pH 9.0 solution and added to 10 mL in a volumetric flask.

Reversed-phase HPLC on a Lichrospher RP-8 Non Endcpd column, with a pore size of 5 μm, i.d. 4.6 mm, and 150 mm length (Alltech, Lexington, KY, USA), and a Perkin-Elmer series 200 HPLC (Waltham, MA, USA) was used to monitor the hydrolytic stability of the targeted peptides. The UV detection was realized at 254 nm with a PerkinElmer series 200 detector (Waltham, MA, USA). The other parameters of the system were the following: an injection volume of 20 μL, room temperature, and 0.70 mL/min flow rate. The gradient was set at 0.0 min at 20% B, 10 min at 100% B, 10 to 13 min at 100% B, 13 to 14 min at 20% B, and 16.5 min at 20% B with mobile phases A: Acetonitrile/water/TFA—5:95:0.1; B: Acetonitrile/water/TFA—95:5:0.1.

## 3. Results

### 3.1. Peptide Synthesis and Characterization

**Figure 1:**
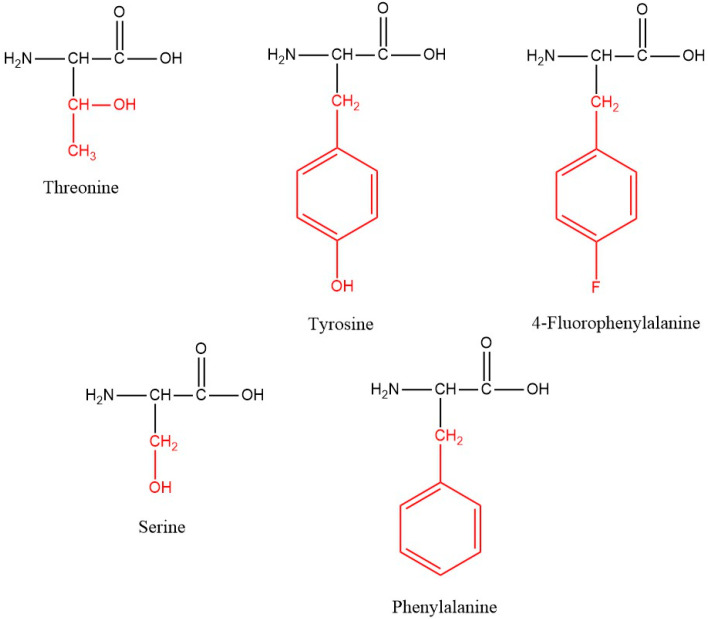
Structures of the amino acids used for modification. The side chains of the amino acids are colored in red.

New C-terminal amide analogues of Temporin A (FLPLIGRVLSGIL-NH_2_) with a general structure FLPLIGRVL-**X_1_**-GIL-NH_2_, where X_1_ = Tyr or Thr, and **X_2_**-LPLIGRVLSGIL-NH_2_, where X_2_ denotes Tyr or Phe(4-F), were synthesized (Figure 1).

The physicochemical characteristics of the novel compounds are outlined in Table 1.

### 3.2. Safety Testing and Antiproliferative Activity

#### 3.2.1. Safety Testing

**Figure 2:**
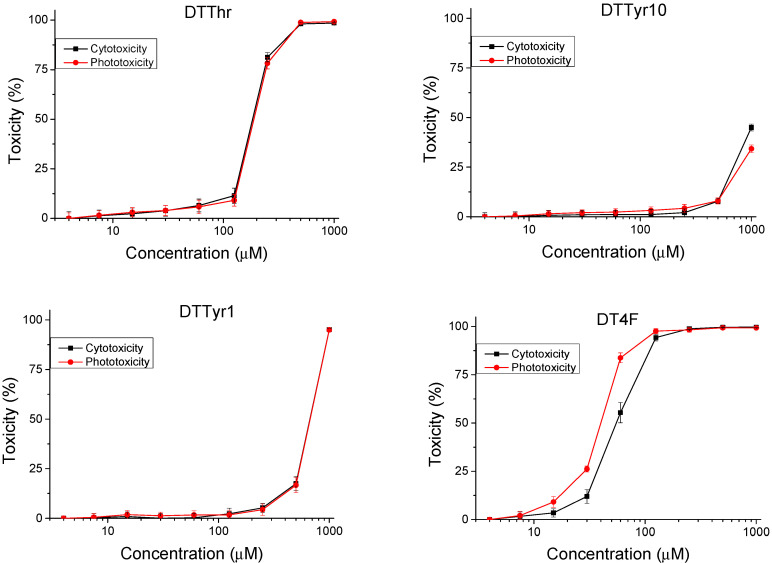
Cytotoxicity/phototoxicity of peptide analogues determined in BALB 3T3 cells; *n* = 6.

An in vitro 3T3 NRU test was used to determine the safety of the peptide analogues. Cytotoxicity is presented as % of the negative control. The resulting dose–response curves are presented in Figure 2. The 50% cytotoxic concentration (CC_50_ value) was calculated through nonlinear regression analysis (Table 2). We observed a low level of toxicity on the peptide analogues DTTyr10 and DTTyr1, with average values of CC_50_ > 1000 and 668.98 ± 13.39 µM, respectively. Significantly (*p* < 0.001) higher cytotoxicity was observed for the other two peptides (DTThr and DT4F), with CC_50_ = 183.36 ± 2.91 µM and 55.41 ± 4.64 µM, respectively. To evaluate the phototoxicity of the peptide analogues, the photo-irritation factor (PIF) was used. The PIF values were calculated using the formula PIF = (CC_50_ − Irr)/(CC_50_ + Irr). The calculated PIF values were lower than 2 for all tested peptides, which means a high level of photo safety.

#### 3.2.2. Antiproliferative Activity

**Figure 3:**
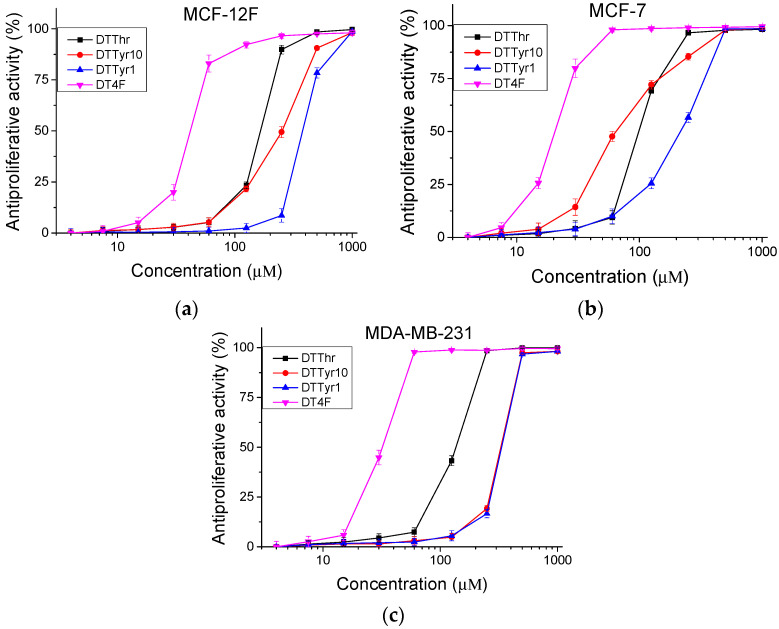
Antiproliferative activity of peptide analogues determined in (**a**) non-tumorigenic MCF-12F cells, (**b**) tumor cell lines MCF-7, and (**c**) MDA-MB-231; n = 6.

The peptide analogues were studied for antiproliferative activity with an MTT assay. Cells were incubated with the tested peptides for 72 h. The obtained results are shown in Figure 3. The mean IC_50_ values and selectivity index were calculated and are presented in Table 3. In the MCF-12F cell line, the highest antiproliferative effect was observed for peptides DTThr (IC_50_ = 165.09 ± 2.21 µM) and DT4F (IC_50_ = 41.78 ±1.64 µM). Similar results were obtained for the MDA-MB-231 cell line. In contrast, for the MCF-7 cell line, a significantly higher (*p* < 0.001) antiproliferative activity of the investigated peptide analogues was detected. In MCF-7 cells, the peptides DTTyr10 and DT4F were the most active, with IC_50_ = 64.51 ± 3.93 µM and 20.33 ± 0.6 µM, respectively. The same peptides also exhibited the highest selectivity towards MCF-7 cells (SI = 3.9 and 2.06, respectively).

### 3.3. Antimicrobial Activity

#### 3.3.1. Disk Diffusion Method

In order to determine the susceptibility of the testing strains to the novel peptides, the disk diffusion method was employed. Thus, 100 μL of the microbial cultures was layered on sterile medium plates. Sterile paper disks were soaked with 20 μL of peptide solution with concentrations of 1.4 mg/mL or 10 mg/mL. Lastly, the disks were put on sterile plates. Studies were conducted in triplicate for each concentration level. The diameter of the inhibition zones in mm was the average value. Additionally, the standard deviation (SD) was calculated. The obtained results for the tested strains are summarized in Table 4, Table 5 and Table 6.

The percentage of inhibition for each synthesized analogue was calculated for all strains in both concentration levels, using the inhibition zone of the antibiotic as a reference point for 100% inhibition. Taking into account the obtained results, the percentage for each peptide analogue was extrapolated. Table 6 provides a summary of the values.

#### 3.3.2. Minimal Inhibitory Concentration (MIC)

The following analyses were carried out to ascertain the MICs of samples. The microbial cultures were added to the 96-well plates containing peptide solutions ranging in concentration from 0 to 320 µg/mL. After a 24-h incubation period, the absorbance at 630 nm was monitored for each plate. The experiment was performed in triplicate (Tables S1–S10). Table 7 summarizes the obtained MIC values.

Tests for MBC values of the novel peptides were also conducted for strains *B. subtilis* 3562, *A. oxydans* 9333, and *P. aeruginosa* 3700. The obtained data showed that the novel peptides did not exhibit bactericidal activity in the tested concentration range.

### 3.4. Hydrolytic Stability

The hydrolytic stability of the obtained peptides was evaluated in model systems that mimic three parts of the human organism: stomach (pH 2.0), blood plasma (pH 7.4), and small intestine (pH 9.0).

**Figure 4:**
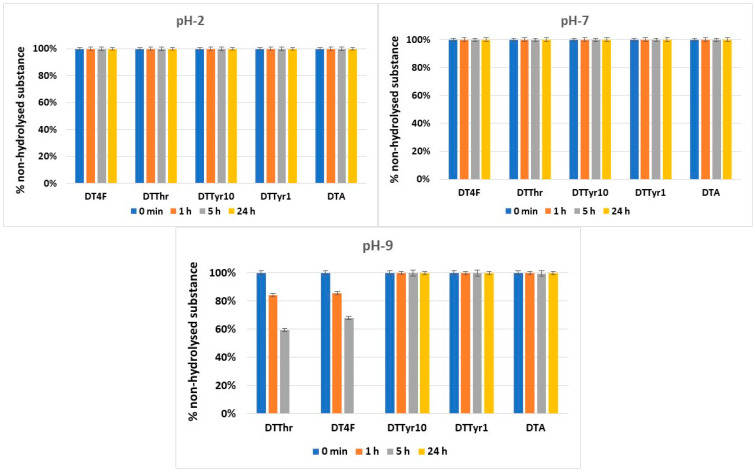
Hydrolysis of the targeted peptides over a 24 h period at three tested pH values.

Most of the newly synthesized peptides were stable in the model systems used for hydrolytic stability investigation, except for DTThr and DT4F in pH 9.0. The dynamics of the hydrolysis during the 24-h period are presented in Figure 4.

## 4. Discussion

Following the standard solid phase peptide synthesis, i.e., the Fmoc/O^t^-Bu approach, the newly designed Temporin A analogues with specific modifications in positions 1 or 10 were synthesized. These specific modifications were performed to examine the influence of the hydroxyl function (primary, secondary, or aromatic) in the molecule, as well as fluorine incorporation, on antimicrobial and antiproliferative activity and hydrolytic stability.

The modification of the parent peptide F^1^LPLIGRVLS^10^GIL-NH_2_ (DTA) at positions 1 or 10 with Tyr (DTTyr10, DTTyr1) resulted in a significant reduction in the toxicity—six and more than ten times, respectively. Thus, the assumption could be made that an aromatic ring together with a phenol type hydroxyl function is preferable for lower toxicity than aliphatic (Ser^10^) or a lack of OH group (Phe^1^) in the parent DTA molecule. In contrast, the incorporation of fluorine in the primary structure of the peptide by the substitution of proteinogenic Phe in position 1 with fluorinated Phe(4-F) (DT4F) resulted in a two-fold increase in the cytotoxicity compared to the parent peptide (Figure 2 and Table 2). However, none of the newly designed Temporin A analogues exhibited phototoxicity.

All newly synthesized analogues of Temporin A were tested against two model Gram(+) bacterial strains, two model Gram(−) bacterial strains, and one fungal strain, using disk diffusion and broth dilution methods. The chosen strains represent some of the most common microorganisms responsible for various infections. For example, *E. coli* causes urinary tract and bloodstream infections [14], *P. aeruginosa* is the reason for respiratory tract infections [15], and *C. albicans* is responsible for mucosal infections [16]. While *B. subtilis* is mostly considered to be non-pathogenic, there are some reports of infections of the central nervous system related to this bacterium [17]. It also becomes pathogenic in combination with other *Bacillus* strains [18]. Clinical reports of *Arthrobacter* species in humans and animals indicate that they may function as opportunistic pathogens, especially in immunocompromised hosts [19]. Overall, the obtained results by both techniques showed that the novel peptides did not exhibit activity against *E. coli* 8785. None of the examined compounds showed antifungal potential either.

Although the fluorinated DT4F was two-fold more cytotoxic compared to the parent molecule, the disk diffusion test results revealed that this molecule was the most active towards the two Gram(+) strains, *B. subtilis* 3562 and *A. oxydans* 9333. DT4F was also active against Gram(−) *P. aeruginosa* 3700, with the greatest inhibition percentage between 33% and 51% at a lower concentration (1.4 mg/mL) and 49% and 68% at a higher dose (10 mg/mL). Thus, when comparing the inhibition zones to those of the parent Temporin A (DTA), the fluorinated one (DT4F) had greater inhibitory potential, especially towards *P. aeruginosa* 3700 [12]. In accordance with the studies of Rosenfeld et al., Mangoni and Shai, and our previous results, the novel compounds were more active towards Gram(+) than Gram(−) strains [12,20,21]. None of the tested new peptides exhibited activity against *E. coli* 8785, which is in correlation with our previous results for a series of Temporin analogues [12]. An interesting observation can be made regarding DTThr activity against *B. subtilis* 3562. It formed an inhibition zone at the lower concentration of 1.4 mg/mL but did not form an inhibition zone at the higher tested concentration of 10 mg/mL. This result strongly correlates with Zapadka et al.’s conclusion that a higher concentration of the peptide could have an adverse effect on stability, thus causing the compounds to become completely inactive or have lower activity [22].

According to the obtained data from the MIC determination, all targeted compounds exhibited bacteriostatic properties against *B. subtilis* 3562, *A. oxydans* 9333, and *P. aeruginosa* 3700. Regarding the strains *E. coli* 8785 and *C. albicans* 74, only the parent peptide DTA exhibited bacteriostatic and fungistatic properties, respectively. The analogue DT4F appeared to have the lowest MIC values among the novel analogues against *B. subtilis* 3562, *A. oxydans* 9333, and *P. aeruginosa* 3700. When comparing the MIC values of DT4F to those of the parent peptide DTA, the fluorinated one had similar potential to most of the tested strains, or even a greater one. It showed 4 times lower MIC than that of DTA (320 µg/mL) towards *P. aeruginosa* 3700. A very interesting observation was made about the DTTyr10 analogue, which had no inhibition zones towards any of the tested strains but had an MIC of 320 µg/mL for *B. subtilis* 3562, *A. oxydans* 9333, and *P. aeruginosa* 3700. A possible explanation of the observed results are the differences between the two methods regarding the interaction of the peptide with the media and bacterial cells [23].

When comparing the effect of the replacement of the Ser residue in position 10 with Tyr and Thr, the novel peptide, containing the spatially more constrained Thr residue, had better antibacterial properties against *B. subtilis* 3562 and the same activity towards the other Gram(+) strain, *A. oxydans* 9333, and the Gram(−) bacteria *P. aeruginosa* 3700. Furthermore, the inhibition zones in the disk diffusion method obtained after treatment with DTThr showed that the alkyl lateral chain of Thr had a better effect than the aromatic and more hydrophobic side chain of Tyr.

The phospholipid bilayer of normal mammalian cells is characterized by the presence of many zwitterion structures such as phosphatidylethanolamine and phosphatidylcholine. They all determine a neutral total charge and make these cells less attractive to cationic antimicrobial peptides [24]. Taking into account this fact, many scientific groups have tested peptides with proven antimicrobial activities for possible antiproliferative effects [25,26,27]. Thus, herein, the antiproliferative activity of newly synthesized molecules was tested against an in vitro model of luminal type A (MCF-7 cells) and basal B-type (MDA-MB-231 cells) breast cancers. The non-tumorigenic cell line MCF-12F was used as a healthy tissue model. The mean IC_50_ values were similar in the non-tumorigenic cell line and the basal-type breast cancer for all peptides. This means that the tested peptides did not have selectivity according to the basal-type breast cancer. In contrast, in the luminal type of cancer, significantly lower IC_50_ values were observed. The highest selectivity was detected for the peptide DTTyr10 against the MCF-7 cells, namely, SI = 3.9. Moreover, at a concentration of 64.51 µM, the compound DTTyr10 caused 50% inhibition of the proliferation of MCF-7 cells, while no antiproliferative activity was observed in non-tumorigenic cells (MCF-12F) at the same concentration. Thus, the obtained results indicate that DTTyr10 treatment has the potential to be effective against luminal breast cancer. The use of halogenated amino acids, especially fluorinated ones, for therapeutics has been an area of immense interest due to their antitumor activity [28]. The positive effect of the fluorine atom on antiproliferative activity is also confirmed in our studies with the Temporin A analogue DT4F. This molecule had good selectivity according to luminal type A (MCF-7 cells) breast cancer, with a selective index a little higher than those of the parent molecule.

One of the most crucial characteristics of novel molecules for use in medicinal practice is their hydrolytic stability. Taking into account this fact, the newly synthesized peptides were evaluated for stability in three model systems that replicate various parts of the human body, namely, pH 2 (stomach), pH 7.4 (blood plasma), and pH 9 (small intestine), for a period of 24 h. The hydrolytic model systems included pepsin and trypsin enzymes at concentrations of 0.5 mg/mL and 0.1 mg/mL, respectively, in a ratio of peptide/pepsin 1:20 and peptide/trypsin 1:100 [27]. The concentration of the tested compounds was 1.0 mg/mL. The conducted experiments showed that all peptides were completely stable at pH 2 and 7 for a tested period of 24 h. At pH 9, the peptides including Thr in position 10 (DTThr) and fluorinated Phe in position 1 (DT4F) underwent complete hydrolysis in 24 h. The results obtained strongly correlate with our previous results obtained with another group of anticancer peptides, which were analogues of natural somatostatin [29]. The results reveal that the introduction of halogen atoms, i.e., chlorine or fluorine, leads to a decrease in hydrolytic stability in basic pH stronger for chlorine and weaker for fluorine atoms. On the other hand, analogues containing a more hydrophobic aromatic lateral chain of Tyr and Phe show good stability for 24 h at the three tested pH values.

## 5. Conclusions

Finally, the obtained structure–activity relationship data show that the most promising compound in the tested series was FLPLIGRVL-Y-GILNH_2_ (DTTyr10) where the Ser residue in position 10 was replaced by a more hydrophobic OH-containing Tyr residue. The compound had very good antiproliferative activity against luminal breast cancer (MCF-7) with a high SI = 3.9. Moreover, at a concentration of 64.51 µM, DTTyr10 caused 50% inhibition of the proliferation of MCF-7 cells, while no antiproliferative activity was observed against non-tumorigenic cells (MCF-12F) at the same concentration. In addition, this peptide was not cyto- and phototoxic and completely hydrolytically stable in all tested model pH systems. Unfortunately, this analogue showed a bacteriostatic effect at a relatively high MIC of 320 µg/mL, which ultimately means that it is not possible to use it as a combined antiproliferative and antimicrobial agent. On the contrary, the analogue of Temporin A containing fluorinated Phe in position 1 (DT4F) had the highest antiproliferative effect against both tested tumor cell lines, namely, MCF-7 and a highly aggressive, invasive, and poorly differentiated triple-negative breast cancer, MDA-MB-231; it also had good antibacterial properties at the lowest MIC 80 µg/mL, but it was more cyto- and phototoxic than the parent DTA molecule. In addition, it was not stable at pH 9 for 24 h period. This result is in full correlation with previously published results that the introducing of fluorine in the peptide molecule leads to better activity with lower hydrolytic stability [29].

## Figures and Tables

**Table 1:** Structure using one-letter amino acid codes, molecular formula, and analytical data from the HPLC-MS analysis (Figure S1), optical rotation determination, and melting point determination of the newly synthesized peptides.

No	Code	Structure	Molecular Formula	MM_exact_ g/mol	[M + H]^+^ Observed g/mol	[M + Na]^+^ Observed g/mol	RT min	α_D_^20^ [°] **	M.p. [°C]
1	DTA *	FLPLIGRVL-**S-**GILNH_2_	C_68_H_117_N_17_O_14_	1395.90	1397.00	1418.95	4.513	−38	158 ± 2
2	DTThr	FLPLIGRVL-**T-**GILNH_2_	C_69_H_119_N_17_O_14_	1409.91	1410.75	1432.70	4.486	−40	135 ± 1
3	DTTyr10	FLPLIGRVL-**Y-**GILNH_2_	C_74_H_121_N_17_O_14_	1471.93	1472.70	1494.70	4.712	−38	145 ± 1
4	DTTyr1	**Y-**LPLIGRVLSGILNH_2_	C_68_H_117_N_17_O_15_	1411.89	1412.60	-	4.177	−58	123 ± 2
5	DT4F	**Phe**(**4F**)**-**LPLIGRVLSGILNH_2_	C_68_H_116_FN_17_O_14_	1413.89	1415.05	1437.10	4.177	−64	141 ± 1

* Data are already published in Dimitrova et al. [12]; ** methanol (c = 1).

**Table 2:** Cytotoxicity/phototoxicity in BALB 3T3 cells and values of CC_50_ and PIF.

Compounds	Mean CC_50_ ± SD (µM)		PIF **
	−Irr	+Irr *	
DTA ***	106.32 ± 4.18	105.40 ± 4.88	1.01
DTThr	183.36 ± 2.91	188.42 ± 3.55	1.0
DTTyr10	>1000	>1000	-
DTTyr1	668.98 ± 13.39	670.76 ± 12.2	1.0
DT4F	55.41 ± 4.64	39.99 ± 0.78	1.4

* Irr—Irradiation; ** PIF—Photo-Irritation Factor. PIF < 2—not phototoxic; 2 < PIF < 5—possible phototoxicity; PIF > 5—phototoxic. *** Data are already published in Dimitrova et al. [12].

**Table 3:** Average IC_50_ values and selectivity index.

Compounds	Mean IC_50_ ± SD (µM)			Selectivity Index (SI) **	
	MCF-12F	MCF-7	MDA-MB-231	MCF-7	MDA-MB-231
DTA *	138.65 ± 8.36	**73.15 ± 3.36**	115.13 ± 4.04	1.90	1.20
DTThr	165.09 ± 2.21	98.57 ± 1.19	136.12 ± 3.61	1.67	1.21
DTTyr10	251.44 ± 11.81	**64.51 ± 3.93**	328.47 ± 2.62	**3.9**	0.77
DTTyr1	317.74 ± 9.79	216.37 ± 10.3	333.55 ± 3.72	1.75	1.13
DT4F	41.78 ± 1.64	**20.33 ± 0.60**	32.05 ± 1.43	**2.06**	1.3

* Data are already published in Dimitrova et al. [12]. ** SI = IC_50_ (normal cell line)/IC_50_ (tumor cell line).

**Table 4:** Inhibition zones (average value in mm with standard deviation) of synthesized peptides and associated antibiotics against Gram(+) strains *B. subtilis* 3562 and *A. oxydans* 9333.

Code	Structure	*B. subtilis* 3562			*A. oxydans* 9333		
		1.4 mg/mL	10 mg/mL	Chloramphenicol [30 µg/disk]	1.4 mg/mL	10 mg/mL	Gentamicin [10 µg/disk]
DTA *	FLPLIGRVL**S**GILNH_2_	8.8 ± 0.3	8.8 ± 0.8	26	6.8 ± 0.3	9.7 ± 0.6	18
DTThr	FLPLIGRVL**T**GILNH_2_	7.2 ± 0.3	0	24	8	10.5 ± 0.5	21
DTTyr10	FLPLIGRVL**Y**GILNH_2_	0	0	27	0	0	23
DTTyr1	**Y**LPLIGRVLSGILNH_2_	0	0	28	10	10	22
DT4F	**Phe**(**4F**)LPLIGRVLSGILNH_2_	8.2 ± 0.3	9.7 ± 0.3	24	10	12.8 ± 0.3	20

* Data are already published in Dimitrova et al. [12].

**Table 5:** Inhibition zones (average value in mm with standard deviation) of synthesized peptides and associated antibiotics against Gram(−) strains *E. coli* 8785 and *P. aeruginosa* 3700.

Code	Structure	*E. coli* 8785			*P. aeruginosa* 3700		
		1.4 mg/mL	10 mg/mL	Gentamicin [10 µg/disk]	1.4 mg/mL	10 mg/mL	Gentamicin [10 µg/disk]
DTA *	FLPLIGRVL**S**GILNH_2_	0	0	18	7.8 ± 0.3	9.3 ± 0.6	17
DTThr	FLPLIGRVL**T**GILNH_2_	0	0	17.5	0	0	17
DTTyr10	FLPLIGRVL**Y**GILNH_2_	0	0	16.5	0	0	18
DTTyr1	**Y**LPLIGRVLSGILNH_2_	0	0	17	0	0	16
DT4F	**Phe**(**4F**)LPLIGRVLSGILNH_2_	0	0	17	9.2 ± 0.3	12.3 ± 0.6	18

* Data are already published in Dimitrova et al. [12].

**Table 6:** Peptide analogues’ percentage-based inhibition of test microorganisms compared to the chosen antibiotics. The antibiotics are taken with 100% inhibition.

Strain		*B. subtilis* 3562		*A. oxydans* 9333		*P. aeruginosa* 3700	
Code	Concentration	1.4 mg/mL	10 mg/mL	1.4 mg/mL	10 mg/mL	1.4 mg/mL	10 mg/mL
1	DTA *	33.8	33.8	37.8	53.9	45.9	54.7
2	DTThr	30.0	0.0	36.4	47.7	0.0	0.0
3	DTTyr10	0.0	0.0	0.0	0.0	0.0	0.0
4	DTTyr1	0.0	0.0	47.6	42.9	0.0	0.0
5	DT4F	32.8	48.5	50.0	64.0	51.1	68.3

* Data are already published in Dimitrova et al. [12].

**Table 7:** MIC values of Temporin A novel analogues [µg/mL].

	Code	Structure	*B. subtilis* 3562	*E. coli* 8785	*A. oxydans* 9333	*P. aeruginosa* 3700	*C. albicans* 74
1	DTA *	FLPLIGRVL**S**GILNH_2_	80 µg/mL	320 µg/mL	80 µg/mL	320 µg/mL	320 µg/mL
2	DTThr	FLPLIGRVL**T**GILNH_2_	160 µg/mL	NI **	320 µg/mL	320 µg/mL	NI **
3	DTTyr10	FLPLIGRVL**Y**GILNH_2_	320 µg/mL	NI **	320 µg/mL	320 µg/mL	NI **
4	DTTyr1	**Y**LPLIGRVLSGILNH_2_	160 µg/mL	NI **	320 µg/mL	320 µg/mL	NI **
5	DT4F	**Phe**(**4F**)LPLIGRVLSGILNH_2_	80 µg/mL	NI **	160 µg/mL	80 µg/mL	NI **

* Data are already published in Dimitrova et al. [12]. ** NI—no inhibition observed.

## Data Availability

Small quantities of all substances are available from authors from the University of Chemical Technology and Metallurgy.

## Supplementary Materials

The following supporting information can be downloaded at: https://www.mdpi.com/article/10.3390/pharmaceutics17040396/s1, HPLC and MS profiles are presented as Figure S1. Raw data for the MIC determination of the tested compounds are provided as Tables S1–S10.
