# Supplementary material for: New Temporin A Analogues Modified in Positions 1 and 10—Synthesis and Biological Studies
Source: Pharmaceutics. 2025 Mar 21;17(4):396. doi: 10.3390/pharmaceutics17040396 (PMC12030253; doi:10.3390/pharmaceutics17040396)

DTA

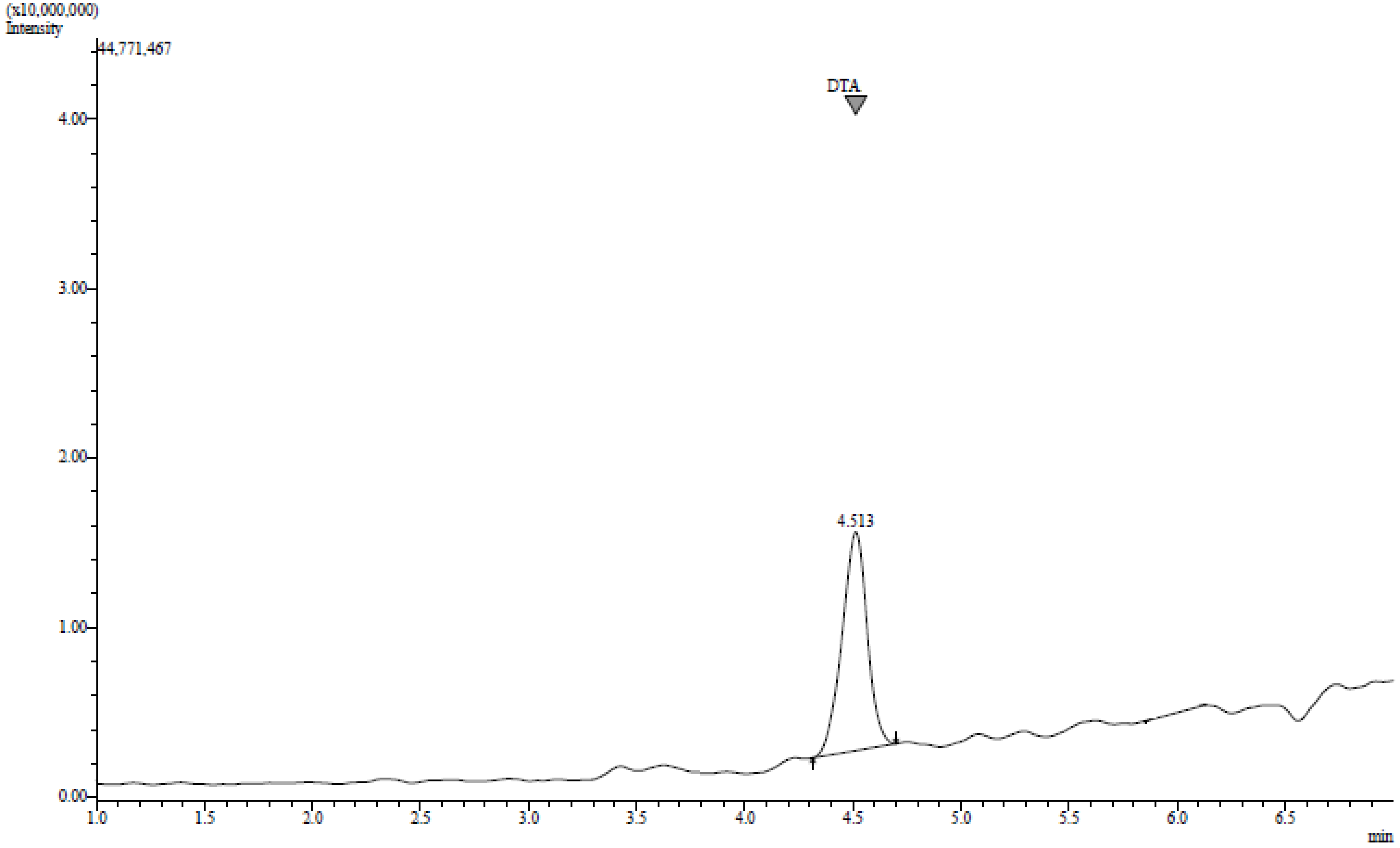

(x10,000,000)
Intensity
44,771,467
4.00
3.00
2.00
1.00
0.00
DTA
4.513
1.0
1.5
2.0
2.5
3.0
3.5
4.0
4.5
5.0
5.5
6.0
6.5
min

ID#:1 R.Time:4.513(Scan#:1355)

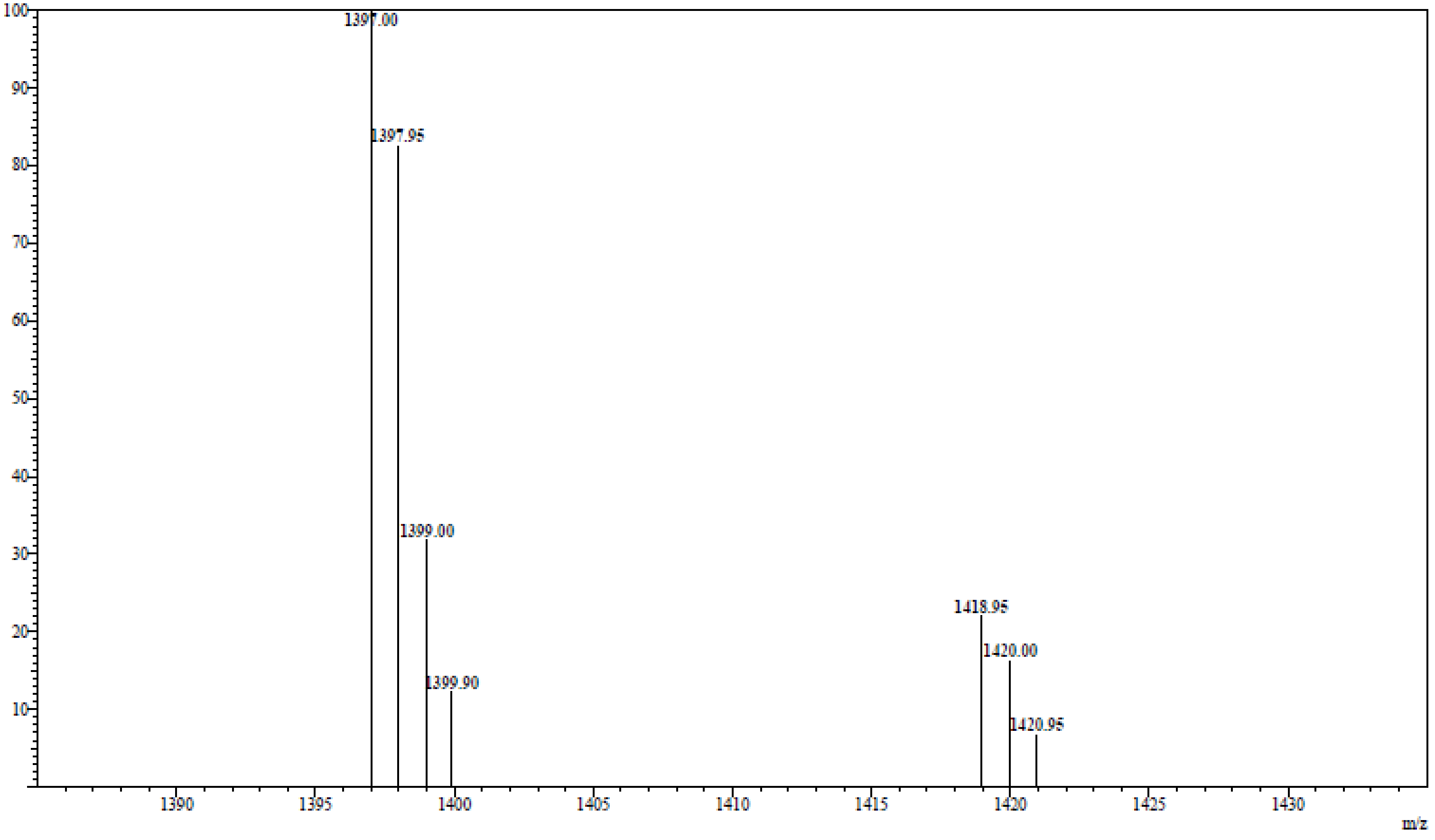

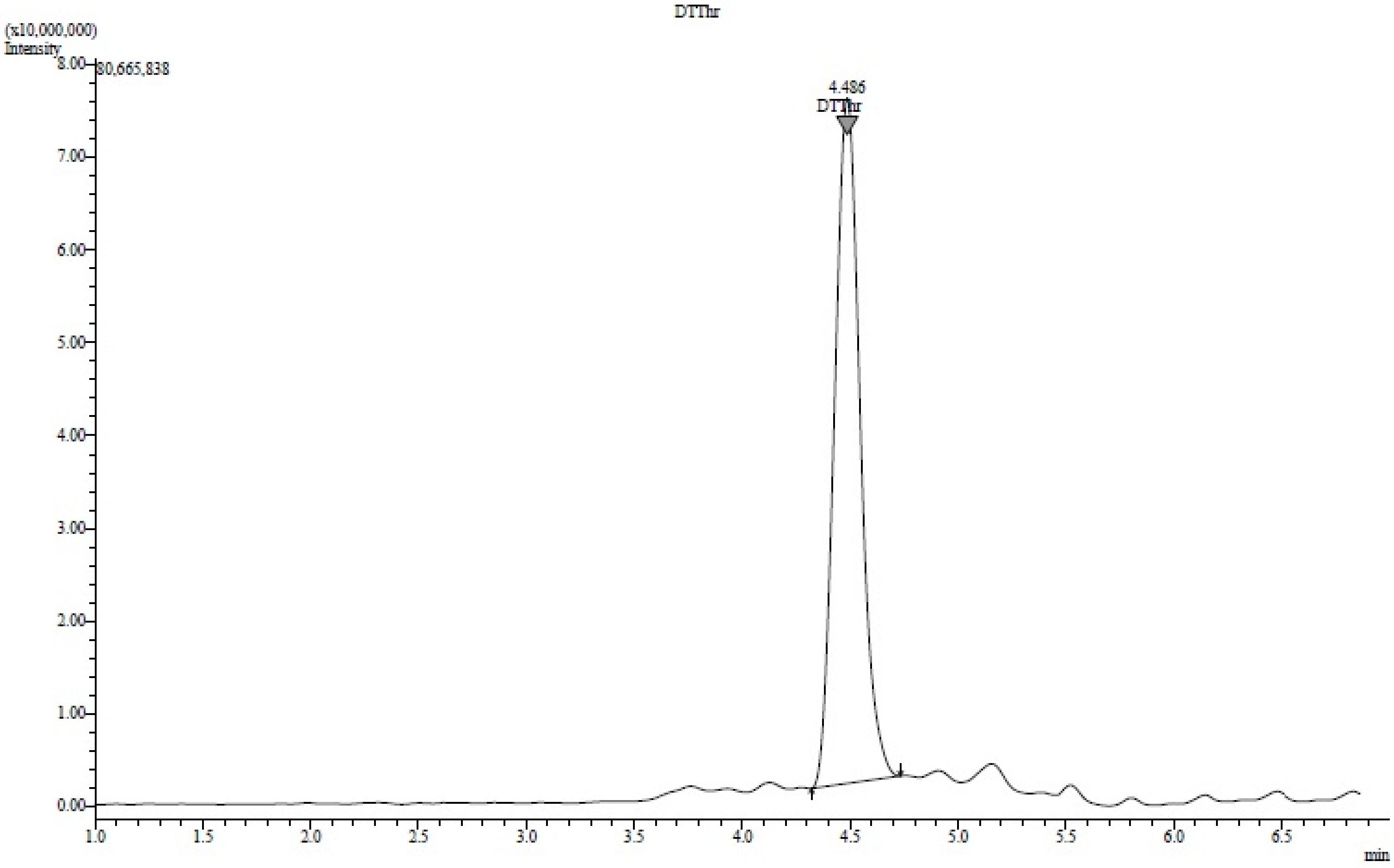
DTThr
(x10,000,000)
Intensity
80,665,838
4.486
DTThr
8.00
7.00
6.00
5.00
4.00
3.00
2.00
1.00
0.00
1.0
1.5
2.0
2.5
3.0
3.5
4.0
4.5
5.0
5.5
6.0
6.5
min

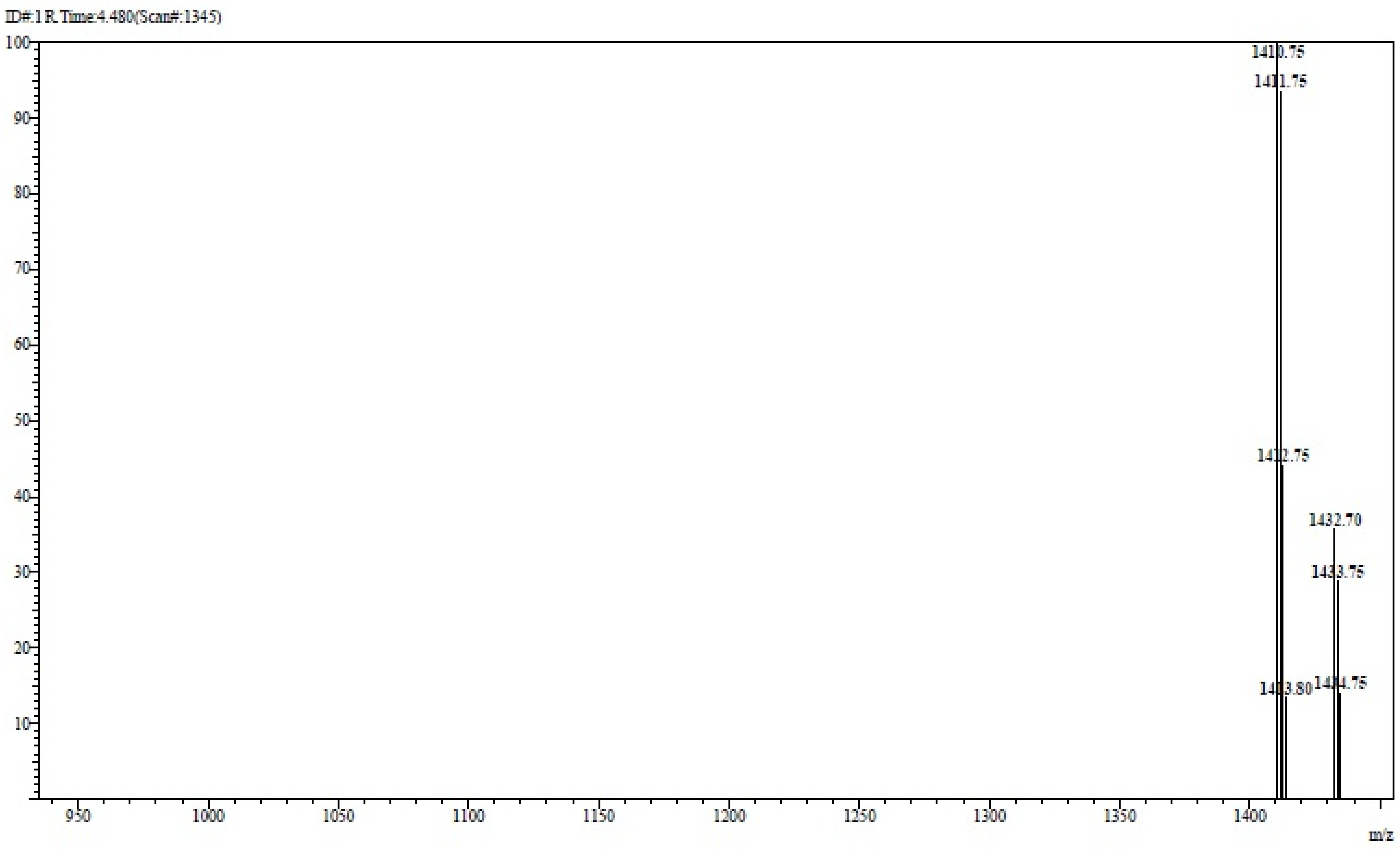

ID#:1 R.Time:4.480(Scan#:1345)
100
90
80
70
60
50
40
30
20
10
1410.75
1411.75
1412.75
1432.70
1433.75
1413.80
1434.75
950
1000
1050
1100
1150
1200
1250
1300
1350
1400
m/z

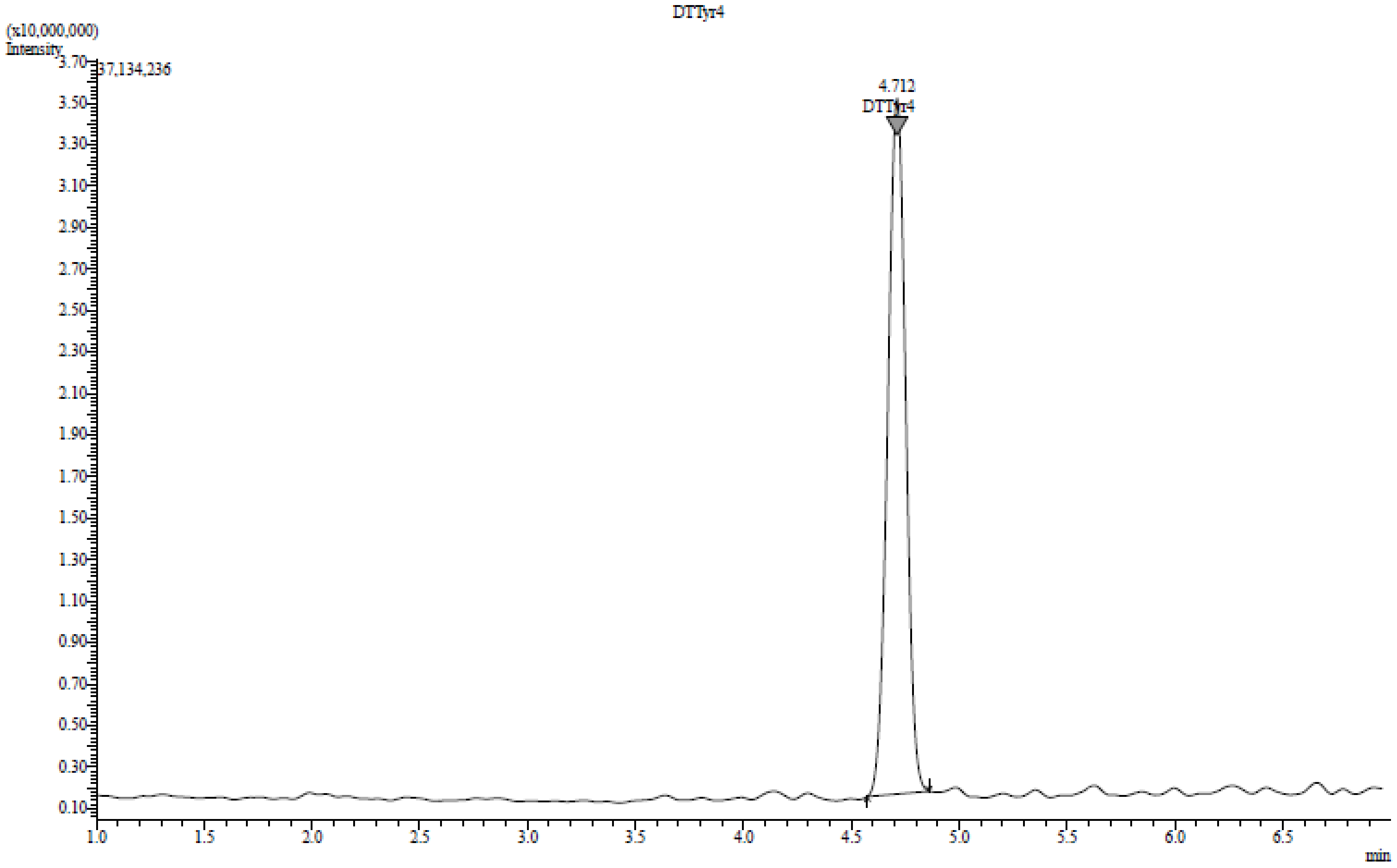

DTTyr4
(x10,000,000)
Intensity
37,134,236
4.712
DTTyr4
3.70
3.50
3.30
3.10
2.90
2.70
2.50
2.30
2.10
1.90
1.70
1.50
1.30
1.10
0.90
0.70
0.50
0.30
0.10
1.0
1.5
2.0
2.5
3.0
3.5
4.0
4.5
5.0
5.5
6.0
6.5
min

MS Spectrum

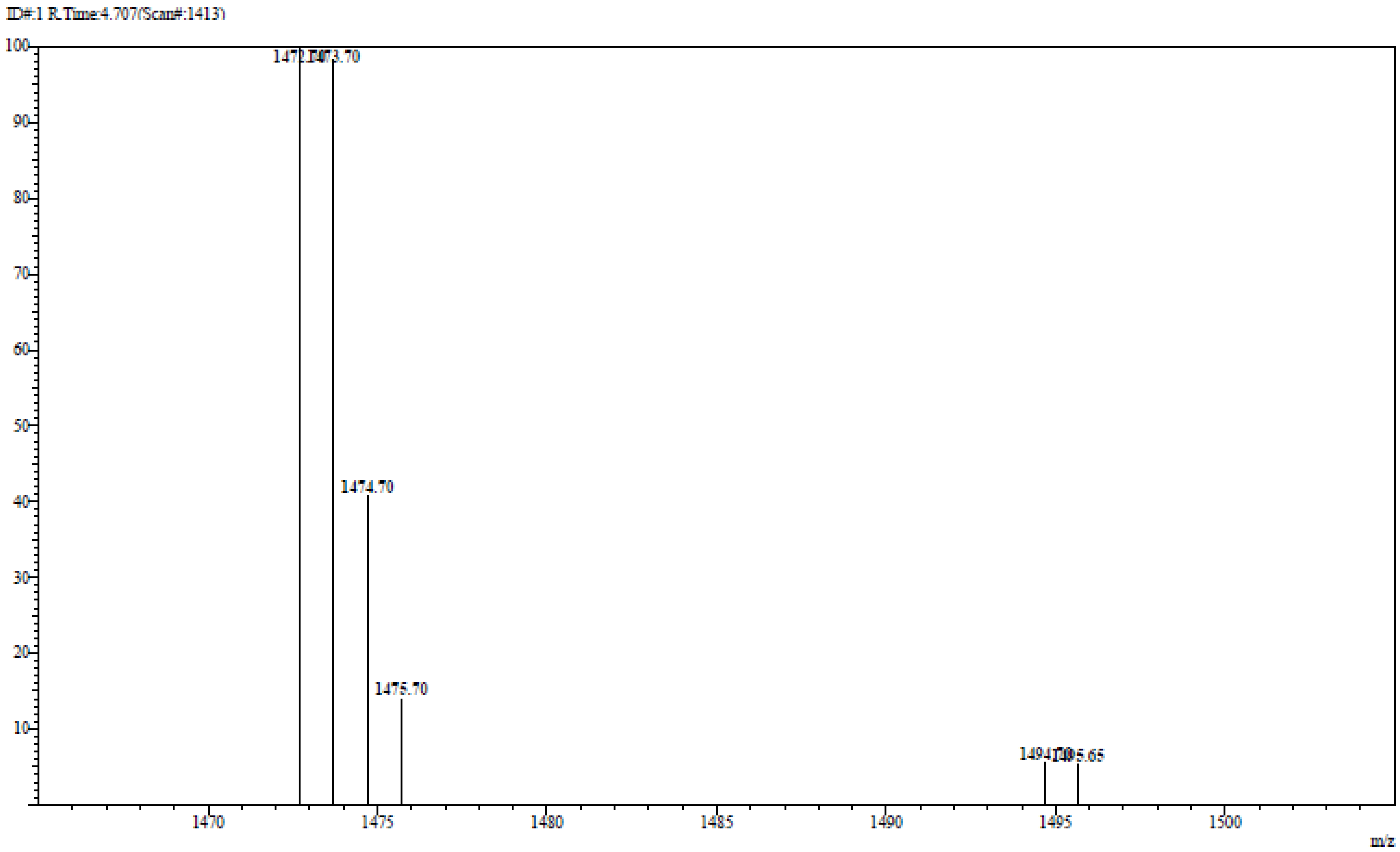

ID#:1 R.Time:4.707(Scan#:1413)
100
90
80
70
60
50
40
30
20
10
1472.70
1473.70
1474.70
1475.70
1494.70
1495.65
1470
1475
1480
1485
1490
1495
1500
m/z

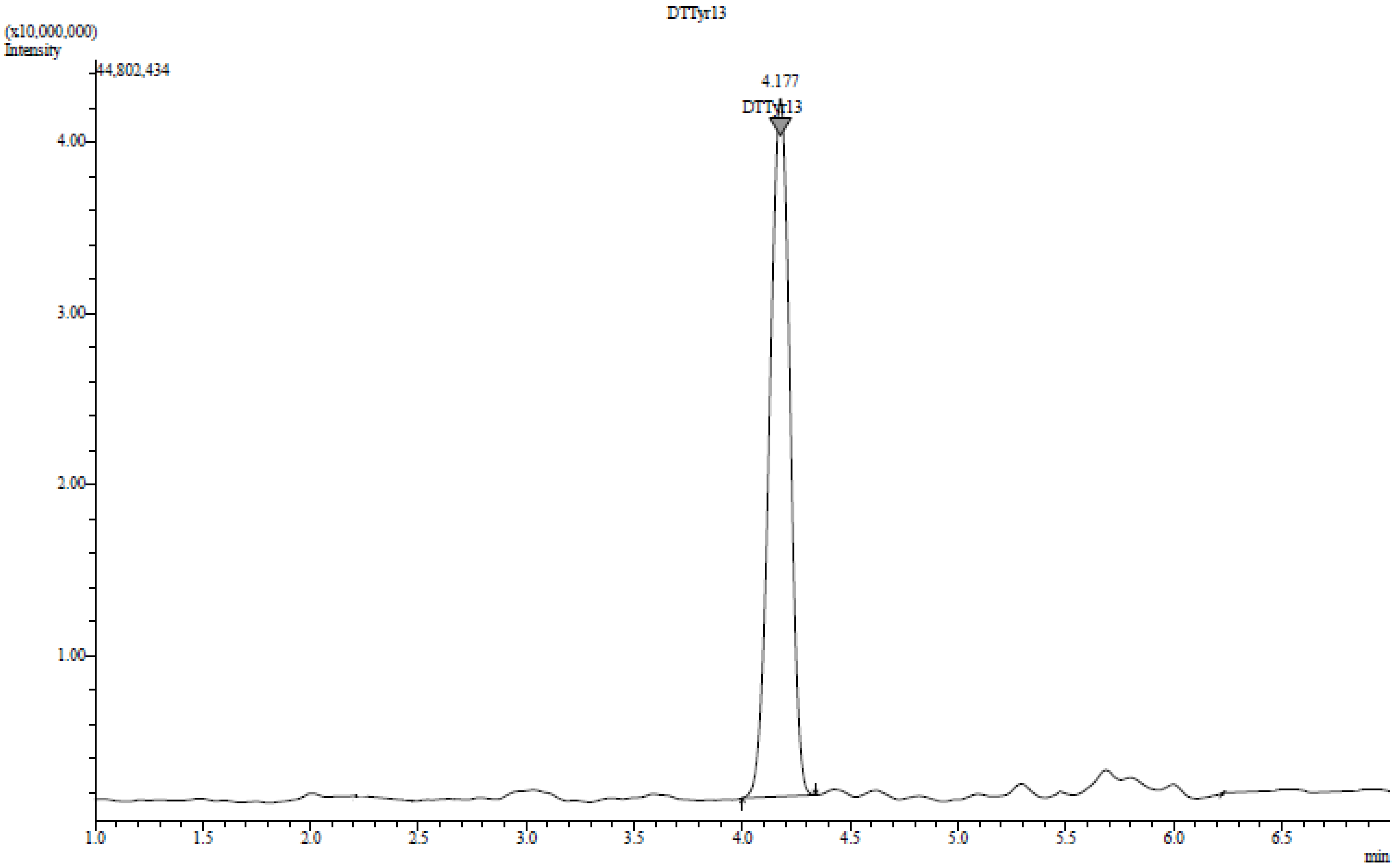

DTTyr13
(x10,000,000)
Intensity
44,802,434
4.177
DTTyr13
4.00
3.00
2.00
1.00
1.0
1.5
2.0
2.5
3.0
3.5
4.0
4.5
5.0
5.5
6.0
6.5
min

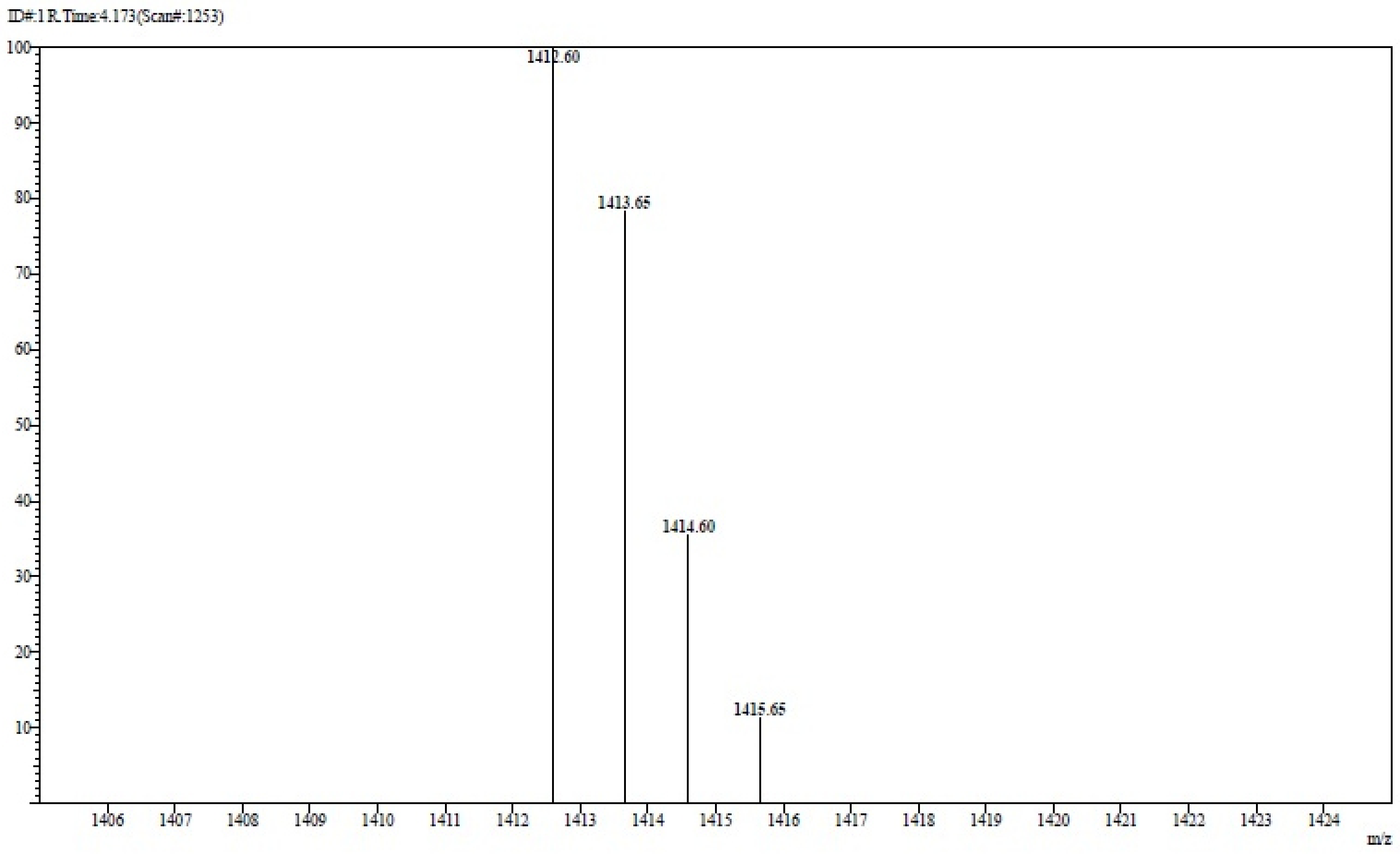
ID#:1 R.Time:4.173(Scan#:1253)
1412.60
1413.65
1414.60
1415.65
100
90
80
70
60
50
40
30
20
10
1406
1407
1408
1409
1410
1411
1412
1413
1414
1415
1416
1417
1418
1419
1420
1421
1422
1423
1424
m/z

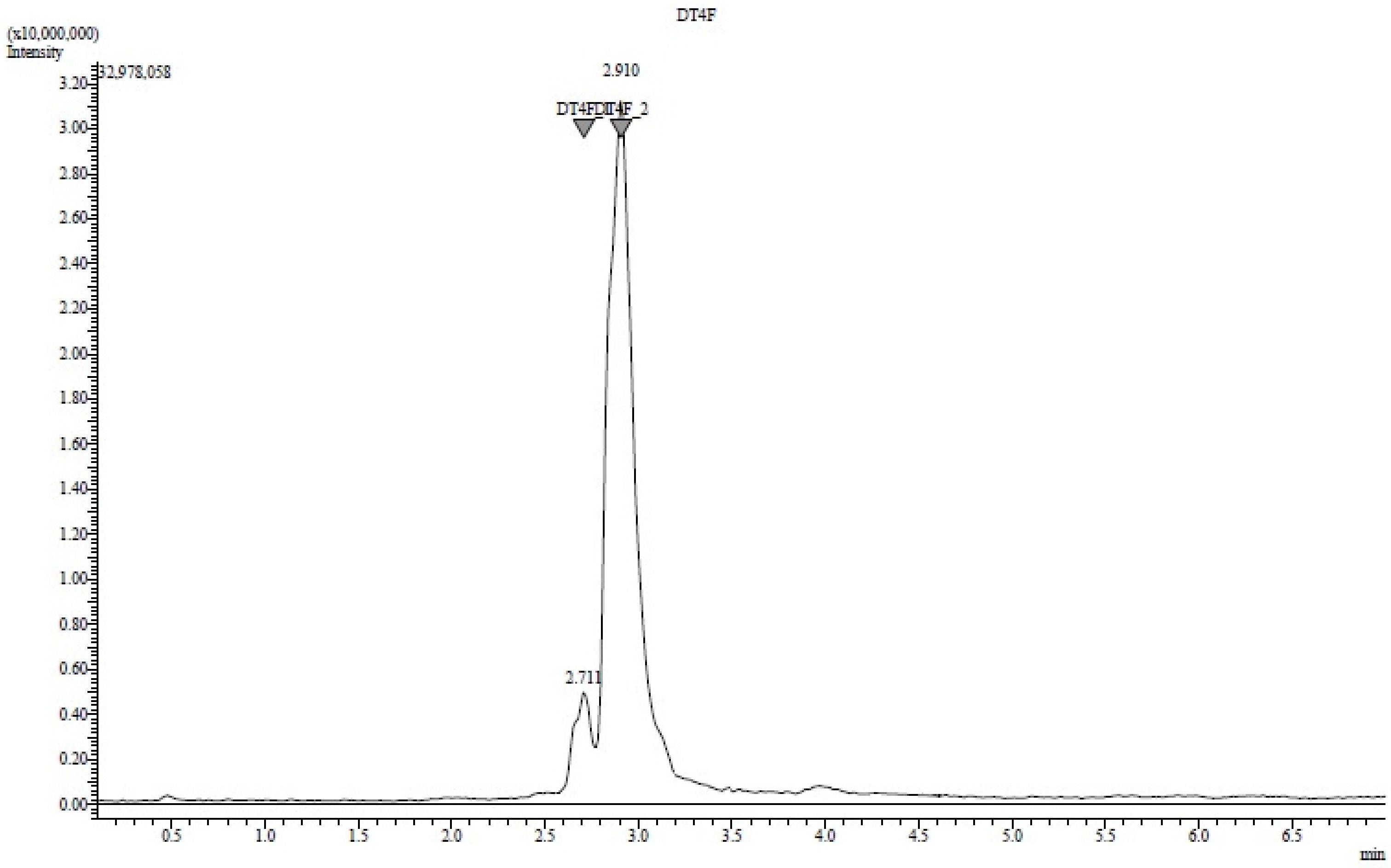

Note: both pics are for the same compound, possible isomers of fluorine atom according to the plane of aromatic ring.

MS Spectrum

ID#:1 R.Time:2.707(Scan#:407)

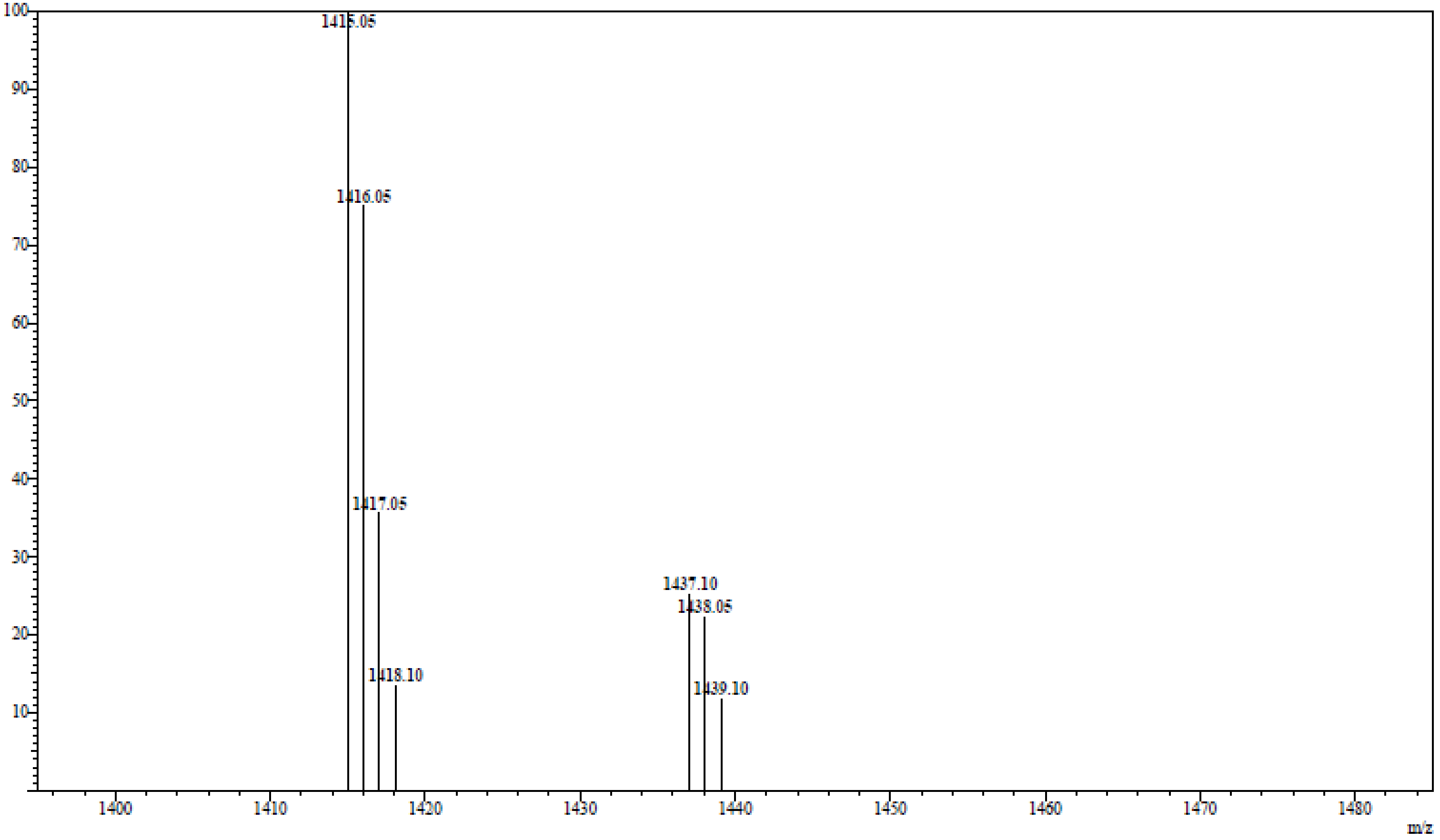

ID#:2 R.Time:2.907(Scan#:437)

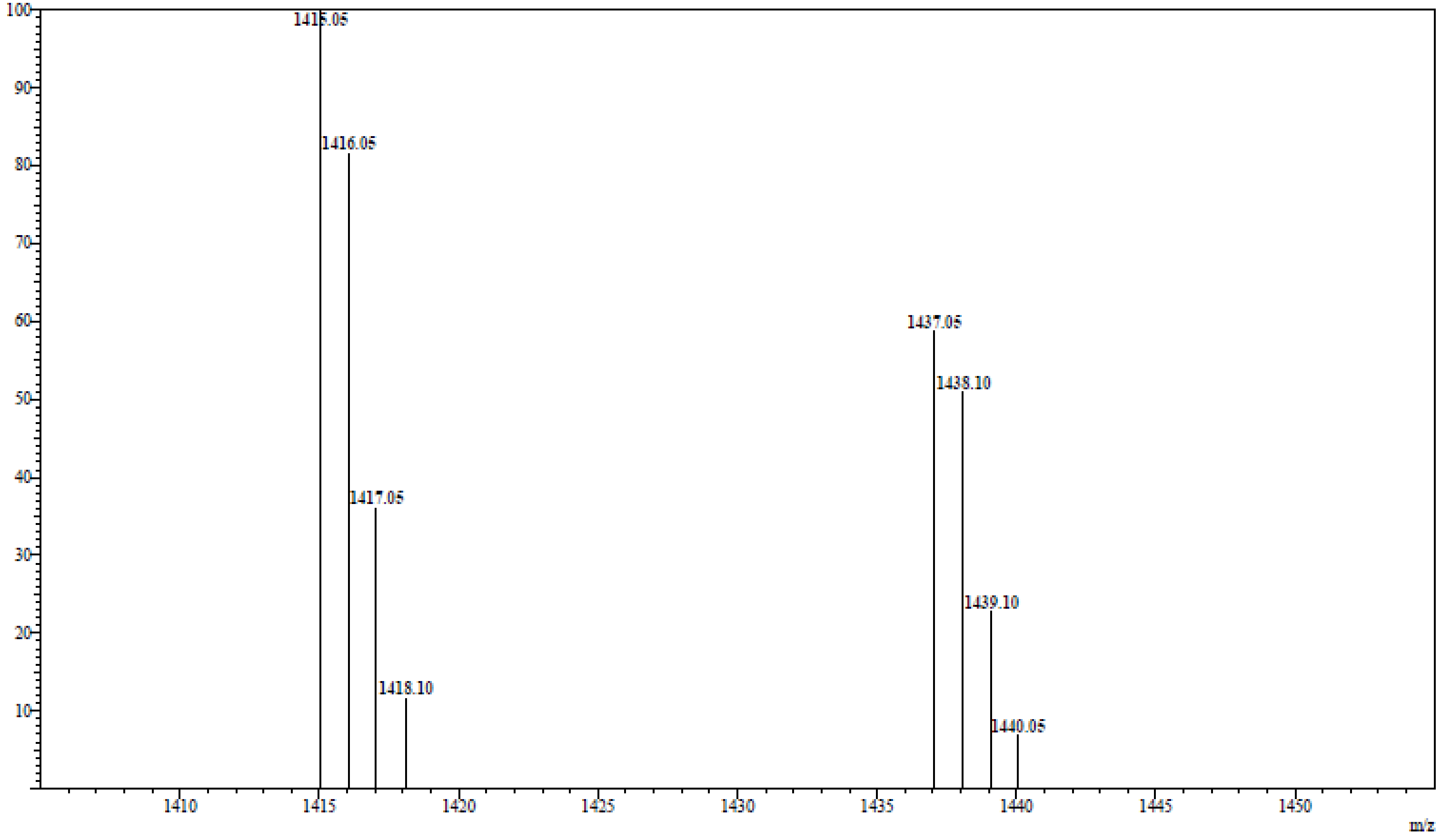

Supplement: Supplementary file 1 [file pharmaceutics-17-00396-s001.zip › Figure S1 - HPLC-MS.pdf]
