# Supplementary material for: New Temporin A Analogues Modified in Positions 1 and 10—Synthesis and Biological Studies
Source: Pharmaceutics. 2025 Mar 21;17(4):396. doi: 10.3390/pharmaceutics17040396 (PMC12030253; doi:10.3390/pharmaceutics17040396)
Supplement: Supplementary file 1 [file pharmaceutics-17-00396-s001.zip › Tables S1-S10.pdf]

Table S1. Raw absorbance data for *Escherichia coli* 8785 at 630 nm for DTA, DTThr, DTTyr4, DTTyr13 and DT4F, where row A represents the positive control (i.e. only strain), as well as 10% EtOH/H2O control and a blank control, i.e. where there is no bacterial strain.

***Escherichia coli* 8785**

0h		DTA			DTThr			DTTyr4			DTTyr13			DT4F		
	Concentration [µg/mL]	*1*	*2*	*3*	*1*	*2*	*3*	*1*	*2*	*3*	*1*	*2*	*3*	*1*	*2*	*3*
**A**	0 (positive control)	*0.0464*	*0.0412*	*0.0413*	*0.0428*	*0.0438*	*0.04*	*0.0363*	*0.0381*	*0.0434*	*0.047*	*0.052*	*0.0572*	*0.0515*	*0.0588*	*0.0616*
**B**	10	*0.0573*	*0.0504*	*0.0468*	*0.0399*	*0.0425*	*0.0423*	*0.0413*	*0.0382*	*0.0438*	*0.0497*	*0.0465*	*0.0499*	*0.0457*	*0.0624*	*0.055*
**C**	20	*0.0572*	*0.0512*	*0.0501*	*0.0484*	*0.0354*	*0.0482*	*0.0452*	*0.0481*	*0.0478*	*0.0638*	*0.0641*	*0.061*	*0.0614*	*0.0757*	*0.0696*
**D**	40	*0.0532*	*0.0551*	*0.0556*	*0.0456*	*0.0408*	*0.0448*	*0.0508*	*0.0553*	*0.0518*	*0.0598*	*0.0553*	*0.0574*	*0.0618*	*0.0603*	*0.0586*
**E**	80	*0.0819*	*0.0684*	*0.0698*	*0.0486*	*0.0563*	*0.0507*	*0.0677*	*0.07*	*0.0677*	*0.0656*	*0.0607*	*0.056*	*0.0809*	*0.0786*	*0.0724*
**F**	160	*0.0853*	*0.1013*	*0.0955*	*0.062*	*0.0616*	*0.0679*	*0.0848*	*0.1082*	*0.0903*	*0.0679*	*0.0622*	*0.0637*	*0.0866*	*0.1062*	*0.1025*
**G**	320	*0.1061*	*0.104*	*0.0904*	*0.0911*	*0.0894*	*0.1018*	*0.1649*	*0.171*	*0.1684*	*0.0853*	*0.0761*	*0.0855*	*0.1256*	*0.1425*	*0.1409*

24h		DTA			DTThr			DTTyr4			DTTyr13			DT4F		
	Concentration [µg/mL]	*1*	*2*	*3*	*1*	*2*	*3*	*1*	*2*	*3*	*1*	*2*	*3*	*1*	*2*	*3*
**A**	0 (positive control)	*1.1823*	*1.0872*	*1.0953*	*1.1225*	*1.2058*	*1.1913*	*1.1788*	*1.1524*	*1.1791*	*1.2309*	*1.1346*	*1.1977*	*1.14*	*1.1487*	*1.16*
**B**	10	*1.2004*	*1.1671*	*1.1428*	*1.1758*	*1.155*	*1.1401*	*1.1082*	*1.1074*	*1.0851*	*1.164*	*1.1001*	*1.0765*	*1.0757*	*1.1996*	*1.0936*
**C**	20	*1.1797*	*1.0774*	*1.0837*	*1.0971*	*1.0722*	*1.0671*	*1.1815*	*1.1227*	*1.1579*	*1.1786*	*1.1061*	*1.0945*	*1.0852*	*1.1701*	*1.1999*
**D**	40	*1.157*	*1.0849*	*1.1192*	*1.151*	*1.0712*	*1.1033*	*1.0973*	*1.0929*	*1.1191*	*1.1779*	*1.1572*	*1.1019*	*1.0753*	*1.1176*	*1.0892*
**E**	80	*1.1204*	*1.1085*	*1.1017*	*1.2218*	*1.1634*	*1.0841*	*1.1347*	*1.273*	*1.1581*	*1.1388*	*1.1055*	*1.1611*	*0.9989*	*1.0281*	*1.0343*
**F**	160	*1.0912*	*1.0646*	*1.0902*	*1.1129*	*1.0859*	*1.0688*	*1.1361*	*1.151*	*1.1192*	*1.1238*	*1.0879*	*1.0941*	*0.8355*	*0.8906*	*0.846*
**G**	320	*0.5691*	*0.6081*	*0.5523*	*0.9586*	*0.9907*	*0.9966*	*1.1103*	*1.0917*	*1.1692*	*1.016*	*0.9114*	*0.9213*	*0.6616*	*0.6472*	*0.6328*

0h		Control 10% EtOH/H_2O					Control blank				
	Concentration [µg/mL]	*1*	2	3	Average	St.dev.	DTA	DTThr	DTTyr4	DTTyr13	DT4F
**A**	0 (positive control)	*0.0571*	*0.0525*	*0.0502*	0.0533	0.0035	0.0528	0.0264	0.0235	0.0306	0.0314
**B**	10	*0.0591*	*0.066*	*0.0539*	0.0597	0.0061	0.0295	0.0221	0.0239	0.0274	0.0293
**C**	20	*0.0615*	*0.0597*	*0.059*	0.0601	0.0013	0.0301	0.0307	0.0264	0.0275	0.0325
**D**	40	*0.053*	*0.0534*	*0.0613*	0.0559	0.0047	0.0297	0.0246	0.0316	0.0247	0.0327
**E**	80	*0.0585*	*0.0597*	*0.0574*	0.0585	0.0012	0.0426	0.0332	0.0509	0.0305	0.0389
**F**	160	*0.0641*	*0.0594*	*0.0616*	0.0617	0.0024	0.0494	0.0401	0.0875	0.0375	0.0533
**G**	320	*0.0675*	*0.0565*	*0.0572*	0.0604	0.0062	0.0822	0.0656	0.1642	0.0628	0.0931

24h		Control 10% EtOH/H_2O					Control blank				
	**Concentration [µg/mL]**	*1*	*2*	*3*	**Average**	**St.dev.**	**DTA**	**DTThr**	**DTTyr4**	**DTTyr13**	**DT4F**
**A**	0 (positive control)	*1.1295*	*1.2345*	*1.1868*	1.1836	0.0526	0.0702	0.7024	0.838	0.9347	0.8742
**B**	10	*1.1129*	*1.1419*	*1.1307*	1.1285	0.0146	0.857	0.6689	0.8504	0.9264	0.9386
**C**	20	*1.1096*	*1.0843*	*1.158*	1.1173	0.0374	0.9422	0.877	0.9902	0.932	0.7411
**D**	40	*1.1269*	*1.1246*	*1.1364*	1.1293	0.0063	0.0289	0.8788	0.7437	0.5652	0.8013
**E**	80	*1.2267*	*1.1134*	*1.165*	1.1684	0.0567	0.0417	0.0589	0.5278	0.5838	0.0836
**F**	160	*1.1262*	*1.164*	*1.1634*	1.1512	0.0217	0.0459	0.055	0.1006	0.0615	0.0813
**G**	320	*1.1852*	*1.1522*	*1.1819*	1.1731	0.0182	0.0779	0.0706	0.8952	0.0569	0.0978

Table S2. Average values with standard deviation of peptides' absorbance for *Escherichia coli* 8785 at 630 nm.

0h		DTA		DTThr		DTTyr4		DTTyr13		DT4F	
	**Concentration [µg/mL]**	**Average**	**St.dev.**	**Average**	**St.dev.**	**Average**	**St.dev.**	**Average**	**St.dev.**	**Average**	**St.dev.**
**A**	**0** (positive control)	0.0430	0.0030	0.0422	0.0020	0.0393	0.0037	0.0521	0.0051	0.0573	0.0052
**B**	10	0.0515	0.0053	0.0416	0.0014	0.0411	0.0028	0.0487	0.0019	0.0544	0.0084
**C**	20	0.0528	0.0038	0.0440	0.0074	0.0470	0.0016	0.0630	0.0017	0.0689	0.0072
**D**	40	0.0546	0.0013	0.0437	0.0026	0.0526	0.0024	0.0575	0.0023	0.0602	0.0016
**E**	80	0.0734	0.0074	0.0519	0.0040	0.0685	0.0013	0.0608	0.0048	0.0773	0.0044
**F**	160	0.0940	0.0081	0.0638	0.0035	0.0944	0.0122	0.0646	0.0030	0.0984	0.0104
**G**	320	0.1002	0.0085	0.0941	0.0067	0.1681	0.0031	0.0823	0.0054	0.1363	0.0093

24h		DTA		DTThr		DTTyr4		DTTyr13		DT4F	
	**Concentration [µg/mL]**	**Average**	**St.dev.**	**Average**	**St.dev.**	**Average**	**St.dev.**	**Average**	**St.dev.**	**Average**	**St.dev.**
A	0 (positive control)	1.1216	0.0527	1.1732	0.0445	1.1701	0.0153	1.1877	0.0489	1.1496	0.0100
B	10	1.1701	0.0289	1.1570	0.0179	1.1002	0.0131	1.1135	0.0453	1.1230	0.0670
C	20	1.1136	0.0573	1.0788	0.0161	1.1540	0.0296	1.1264	0.0456	1.1517	0.0595
D	40	1.1204	0.0361	1.1085	0.0402	1.1031	0.0140	1.1457	0.0393	1.0940	0.0216
E	80	1.1102	0.0095	1.1564	0.0691	1.1886	0.0740	1.1351	0.0280	1.0204	0.0189
F	160	1.0820	0.0151	1.0892	0.0222	1.1354	0.0159	1.1019	0.0192	0.8574	0.0293
G	320	0.5765	0.0286	0.9820	0.0205	1.1237	0.0405	0.9496	0.0577	0.6472	0.0144

Table S3. Raw absorbance data for *Pseudomonas aeruginosa* 3700 at 630 nm for DTA, DTThr, DTTyr4, DTTyr13 and DT4F, where row A represents the positive control (i.e. only strain), as well as 10% EtOH/H2O control and a blank control, i.e. where there is no bacterial strain.

Pseudomonas aeruginosa 3700

0h		DTA			DTThr			DTTyr4			DTTyr13			DT4F		
	**Concentration [µg/mL]**	**1**	**2**	**3**	**1**	**2**	**3**	**1**	**2**	**3**	**1**	**2**	**3**	**1**	**2**	**3**
A	0 (positive control)	0.0389	0.0393	0.0426	0.0539	0.0556	0.0566	0.0556	0.0519	0.0577	0.0658	0.064	0.0908	0.0813	0.0803	0.0756
B	10	0.0458	0.0428	0.0416	0.0779	0.1555	0.2867	0.0754	0.0756	0.0633	0.0987	0.0886	0.086	0.1007	0.089	0.0998
C	20	0.0742	0.062	0.0636	0.0822	0.0785	0.0777	0.0767	0.0914	0.075	0.114	0.1022	0.1014	0.1165	0.1123	0.1152
D	40	0.0655	0.0602	0.0685	0.0925	0.0852	0.0859	0.1046	0.1008	0.0969	0.1114	0.1137	0.1059	0.1045	0.1219	0.1261
E	80	0.0783	0.0805	0.0828	0.1133	0.1079	0.1063	0.1247	0.1088	0.1097	0.1221	0.1371	0.1427	0.1394	0.1452	0.1366
F	160	0.0831	0.0794	0.071	0.1323	0.1176	0.1098	0.152	0.1358	0.1445	0.1422	0.1256	0.1371	0.1446	0.1459	0.1522
G	320	0.1186	0.0845	0.0769	0.137	0.1278	0.116	0.2089	0.2062	0.2121	0.193	0.1477	0.1381	0.1831	0.1999	0.1943

24h		DTA			DTThr			DTTyr4			DTTyr13			DT4F		
	**Concentration [µg/mL]**	**1**	**2**	**3**	**1**	**2**	**3**	**1**	**2**	**3**	**1**	**2**	**3**	**1**	**2**	**3**
A	0 (positive control)	0.501	0.5671	0.3522	0.3694	0.3561	0.398	0.3693	0.3977	0.4819	0.2747	0.2549	0.2623	0.2996	0.3152	0.3076
B	10	0.6641	0.3234	0.4013	0.3641	0.3862	0.4027	0.3328	0.3875	0.3587	0.3513	0.3993	0.4162	0.3304	0.2982	0.344
C	20	0.5969	0.3372	0.309	0.4056	0.3854	0.4114	0.3529	0.4373	0.3734	0.3775	0.3491	0.3635	0.3399	0.3326	0.3375
D	40	0.6036	0.4295	0.3754	0.3653	0.3686	0.3739	0.3823	0.4108	0.4639	0.3252	0.3412	0.332	0.2698	0.2414	0.3167
E	80	0.5338	0.3749	0.4028	0.3516	0.3175	0.3331	0.2934	0.2904	0.2743	0.3273	0.3078	0.3144	0.1585	0.2919	0.29
F	160	0.4803	0.1657	0.236	0.2585	0.2948	0.2798	0.2868	0.2992	0.2877	0.3192	0.2215	0.2487	0.1345	0.1447	0.1643
G	320	0.0874	0.0571	0.0595	0.1616	0.1552	0.1472	0.1861	0.205	0.2354	0.1622	0.129	0.1239	0.1361	0.1484	0.1408

**0h**		**Control 10% EtOH/H_2O**					**Control blank**				
	**Concentration [µg/mL]**	**1**	**2**	**3**	**Average**	**St.dev.**	**DTA**	**DTThr**	**DTTyr4**	**DTTyr13**	**DT4F**
**A**	0 (positive control)	0.0775	0.0805	0.0763	0.0781	0.0022	0.0228	0.0197	0.0135	0.0143	0.0191
**B**	10	0.089	0.0917	0.0923	0.0910	0.0018	0.0257	0.0185	0.0227	0.0268	0.0149
**C**	20	0.1017	0.1102	0.1115	0.1078	0.0053	0.0432	0.0328	0.033	0.0385	0.0421
**D**	40	0.1124	0.0906	0.1022	0.1017	0.0109	0.0507	0.0301	0.0297	0.0346	0.0408
**E**	80	0.1109	0.0969	0.118	0.1086	0.0107	0.0515	0.0492	0.0552	0.027	0.0734
**F**	160	0.1055	0.1154	0.1071	0.1093	0.0053	0.0537	0.0619	0.103	0.0489	0.0921
**G**	320	0.088	0.0993	0.1066	0.0980	0.0094	0.0882	0.0876	0.1858	0.0882	0.1255

24h		Control 10% EtOH/H_2O					Control blank				
	**Concentration [µg/mL]**	***1***	***2***	***3***	**Average**	**St.dev.**	**DTA**	**DTThr**	**DTTyr4**	**DTTyr13**	**DT4F**
**A**	0 (positive control)	*0.4034*	*0.3858*	*0.3364*	0.3752	0.0347	0.0921	0.1548	0.1424	0.1207	0.1501
**B**	10	*0.4414*	*0.2536*	*0.2603*	0.3184	0.1065	0.0314	0.0695	0.0615	0.0667	0.0628
**C**	20	*0.3466*	*0.3623*	*0.3101*	0.3397	0.0268	0.0484	0.0677	0.396	0.0859	0.0938
**D**	40	*0.2574*	*0.2769*	*0.2537*	0.2627	0.0125	0.0524	0.074	0.0923	0.0932	0.1082
**E**	80	*0.5554*	*0.3007*	*0.4137*	0.4233	0.1276	0.0515	0.0974	0.1259	0.1094	0.1512
**F**	160	*0.3689*	*0.5376*	*0.3577*	0.4214	0.1008	0.0519	0.1046	0.1425	0.1095	0.1478
**G**	320	*0.305*	*0.3079*	*0.3104*	0.3078	0.0027	0.0904	0.0964	0.2039	0.1067	0.1108

Table S4. Average values with standard deviation of peptides' absorbance for *Pseudomonas aeruginosa* 3700 at 630 nm.

0h		DTA		DTThr		DTTyr4		DTTyr13		DT4F	
	**Concentration [µg/mL]**	**Average**	**St.dev.**	**Average**	**St.dev.**	**Average**	**St.dev.**	**Average**	**St.dev.**	**Average**	**St.dev.**
**A**	0 (positive control)	0.0403	0.0020	0.0554	0.0014	0.0551	0.0029	0.0735	0.0150	0.0791	0.0030
**B**	10	0.0434	0.0022	0.1734	0.1055	0.0714	0.0070	0.0911	0.0067	0.0965	0.0065
**C**	20	0.0666	0.0066	0.0795	0.0024	0.0810	0.0090	0.1059	0.0071	0.1147	0.0022
**D**	40	0.0647	0.0042	0.0879	0.0040	0.1008	0.0039	0.1103	0.0040	0.1175	0.0115
**E**	80	0.0805	0.0023	0.1092	0.0037	0.1144	0.0089	0.1340	0.0107	0.1404	0.0044
**F**	160	0.0778	0.0062	0.1199	0.0114	0.1441	0.0081	0.1350	0.0085	0.1476	0.0041
**G**	320	0.0933	0.0222	0.1269	0.0105	0.2091	0.0030	0.1596	0.0293	0.1924	0.0086

24h		DTA		DTThr		DTTyr4		DTTyr13		DT4F	
	**Concentration [µg/mL]**	**Average**	**St.dev.**	**Average**	**St.dev.**	**Average**	**St.dev.**	**Average**	**St.dev.**	**Average**	**St.dev.**
A	0 (positive control)	0.4734	0.1101	0.3745	0.0214	0.4163	0.0586	0.2640	0.0100	0.3075	0.0078
B	10	0.4629	0.1785	0.3843	0.0194	0.3597	0.0274	0.3889	0.0337	0.3242	0.0235
C	20	0.4144	0.1587	0.4008	0.0136	0.3879	0.0440	0.3634	0.0142	0.3367	0.0037
D	40	0.4695	0.1192	0.3693	0.0043	0.4190	0.0414	0.3328	0.0080	0.2760	0.0380
E	80	0.4372	0.0848	0.3341	0.0171	0.2860	0.0103	0.3165	0.0099	0.2468	0.0765
F	160	0.2940	0.1651	0.2777	0.0182	0.2912	0.0069	0.2631	0.0504	0.1478	0.0151
G	320	0.0680	0.0168	0.1547	0.0072	0.2088	0.0249	0.1384	0.0208	0.1418	0.0062

Table S5. Raw absorbance data for *Bacillus subtilis* 3562 at 630 nm for DTA, DTThr, DTTyr4, DTTyr13 and DT4F, where row A represents the positive control (i.e. only strain), as well as 10% EtOH/H2O control and a blank control, i.e. where there is no bacterial strain.

Bacillus subtilis 3562																
**0h**		**DTA**			**DTThr**			**DTTyr4**			**DTTyr13**			**DT4F**		
	**Concentration [μg/mL]**	***1***	**2**	***3***	***1***	**2**	***3***	***1***	**2**	***3***	***1***	**2**	***3***	***1***	**2**	***3***
**A**	0 (positive control)	*0.041*	*0.0552*	*0.0556*	*0.0428*	*0.0438*	*0.04*	*0.0363*	*0.0381*	*0.0434*	*0.0399*	*0.0392*	*0.0363*	*0.0451*	*0.0408*	*0.0415*
**B**	10	*0.0425*	*0.0498*	*0.0585*	*0.0399*	*0.0425*	*0.0423*	*0.0413*	*0.0382*	*0.0438*	*0.0406*	*0.0421*	*0.0398*	*0.0425*	*0.0456*	*0.0421*
**C**	20	*0.0517*	*0.049*	*0.0535*	*0.0484*	*0.0354*	*0.0482*	*0.0452*	*0.0481*	*0.0478*	*0.0379*	*0.041*	*0.0418*	*0.0424*	*0.0455*	*0.0483*
**D**	40	*0.0547*	*0.0503*	*0.0476*	*0.0456*	*0.0408*	*0.0448*	*0.0508*	*0.0553*	*0.0518*	*0.0417*	*0.0389*	*0.0415*	*0.0434*	*0.0457*	*0.0438*
**E**	80	*0.0569*	*0.0608*	*0.0721*	*0.0486*	*0.0563*	*0.0507*	*0.0677*	*0.07*	*0.0677*	*0.0515*	*0.0488*	*0.0513*	*0.0585*	*0.0585*	*0.0606*
**F**	160	*0.0758*	*0.0991*	*0.0705*	*0.062*	*0.0616*	*0.0679*	*0.0848*	*0.1082*	*0.0903*	*0.0545*	*0.0576*	*0.0571*	*0.0771*	*0.0714*	*0.0725*
**G**	320	*0.1066*	*0.0946*	*0.0902*	*0.0911*	*0.0894*	*0.1018*	*0.1649*	*0.171*	*0.1684*	*0.0761*	*0.0736*	*0.0712*	*0.1188*	*0.1078*	*0.1149*

**24h**		**DTA**			**DTThr**			**DTTyr4**			**DTTyr13**			**DT4F**		
	**Concentration [μg/mL]**	***1***	**2**	***3***	***1***	**2**	***3***	***1***	**2**	***3***	***1***	**2**	***3***	***1***	**2**	***3***
**A**	0 (positive control)	*0.7053*	*0.5053*	*0.4784*	*0.3764*	*0.43*	*0.4192*	*0.3983*	*0.4393*	*0.5389*	*0.3106*	*0.364*	*0.3535*	*0.2602*	*0.2683*	*0.2682*
**B**	10	*0.0699*	*0.0628*	*0.0583*	*0.3454*	*0.3142*	*0.3808*	*0.3724*	*0.363*	*0.5582*	*0.4207*	*0.363*	*0.3812*	*0.4557*	*0.5098*	*0.5516*
**C**	20	*0.614*	*0.3453*	*0.5451*	*0.4128*	*0.3019*	*0.414*	*0.3935*	*0.4055*	*0.5619*	*0.3703*	*0.5405*	*0.3207*	*0.3*	*0.4244*	*0.5325*
**D**	40	*0.4167*	*0.3557*	*0.3467*	*0.4697*	*0.4782*	*0.4791*	*0.4054*	*0.4186*	*0.5264*	*0.6072*	*0.3022*	*0.4865*	*0.4342*	*0.4017*	*0.3523*
**E**	80	*0.0496*	*0.052*	*0.0486*	*0.2589*	*0.2491*	*0.2308*	*0.3318*	*0.3232*	*0.5147*	*0.6653*	*0.4615*	*0.4115*	*0.0377*	*0.0366*	*0.0416*
**F**	160	*0.0584*	*0.0547*	*0.0473*	*0.0512*	*0.0538*	*0.0622*	*0.3586*	*0.348*	*0.3764*	*0.4067*	*0.5021*	*0.4642*	*0.054*	*0.0509*	*0.0506*
**G**	320	*0.6393*	*0.536*	*0.5327*	*0.0722*	*0.0652*	*0.0716*	*0.1391*	*0.1469*	*0.1475*	*0.058*	*0.0501*	*0.0515*	*0.0858*	*0.077*	*0.0775*

**0h**		**Control 10% EtOH/H_2O**					**Control blank**				
	**Concentration [μg/mL]**	***1***	**2**	**3**	**Average**	**St.dev.**	**DTA**	**DTThr**	**DTTyr4**	**DTTyr13**	**DT4F**
**A**	0 (positive control)	*0.0391*	*0.0327*	*0.0385*	0.0368	0.0035	0.0075	0.0083	0.0092	0.0095	0.0123
**B**	10	*0.0385*	*0.0375*	*0.0451*	0.0404	0.0041	0.0148	0.0096	0.0132	0.0057	0.0096
**C**	20	*0.0414*	*0.0377*	*0.0404*	0.0398	0.0019	0.0098	0.01	0.0152	0.0079	0.0128
**D**	40	*0.0316*	*0.0313*	*0.0322*	0.0317	0.0005	0.0128	0.014	0.0242	0.014	0.0148
**E**	80	*0.0338*	*0.0379*	*0.0345*	0.0354	0.0022	0.0258	0.0285	0.0474	0.0189	0.0322
**F**	160	*0.0328*	*0.0395*	*0.033*	0.0351	0.0038	0.0449	0.0397	0.0742	0.0265	0.0516
**G**	320	*0.0419*	*0.0384*	*0.0346*	0.0383	0.0037	0.1118	0.0681	0.1409	0.0577	0.0846

24h		Control 10% EtOH/H_2O					Control blank				
	Concentration [µg/mL]	*1*	2	*3*	Average	St.dev.	DTA	DTThr	DTTyr4	DTTyr13	DT4F
**A**	0 (positive control)	*0.5946*	*0.5552*	*0.5231*	0.5576	0.0358	0.0161	0.33	0.1803	0.3096	0.3303
**B**	10	*0.317*	*0.2459*	*0.3421*	0.3017	0.0499	0.0166	0.2275	0.2095	0.1513	0.1806
**C**	20	*0.3838*	*0.3579*	*0.3898*	0.3772	0.0170	0.0245	0.1875	0.2285	0.156	0.0268
**D**	40	*0.4544*	*0.393*	*0.4551*	0.4342	0.0357	0.0161	0.1157	0.2626	0.1449	0.0237
**E**	80	*0.4757*	*0.5109*	*0.5119*	0.4995	0.0206	0.0264	0.0772	0.0521	0.1155	0.0355
**F**	160	*0.5216*	*0.5189*	*0.6379*	0.5595	0.0679	0.038	0.0423	0.0816	0.0322	0.0488
**G**	320	*0.543*	*0.7202*	*0.5895*	0.6176	0.0919	0.0801	0.062	0.1212	0.0609	0.0894

Table S6. Average values with standard deviation of peptides' absorbance for *Bacillus subtilis* 3562 at 630 nm.

0h		DTA		DTThr		DTTyr4		DTTyr13		DT4F	
	Concentration [µg/mL]	Average	St.dev.	Average	St.dev.	Average	St.dev.	Average	St.dev.	Average	St.dev.
**A**	0 (positive control)	0.0506	0.0083	0.0422	0.0020	0.0393	0.0037	0.0385	0.0019	0.0425	0.0023
**B**	10	0.0503	0.0080	0.0416	0.0014	0.0411	0.0028	0.0408	0.0012	0.0434	0.0019
**C**	20	0.0514	0.0023	0.0440	0.0074	0.0470	0.0016	0.0402	0.0021	0.0454	0.0030
**D**	40	0.0509	0.0036	0.0437	0.0026	0.0526	0.0024	0.0407	0.0016	0.0443	0.0012
**E**	80	0.0633	0.0079	0.0519	0.0040	0.0685	0.0013	0.0505	0.0015	0.0592	0.0012
**F**	160	0.0818	0.0152	0.0638	0.0035	0.0944	0.0122	0.0564	0.0017	0.0737	0.0030
**G**	320	0.0971	0.0085	0.0941	0.0067	0.1681	0.0031	0.0736	0.0025	0.1138	0.0056

24h		DTA		DTThr		DTTyr4		DTTyr13		DT4F	
	Concentration [µg/mL]	Average	St.dev.	Average	St.dev.	Average	St.dev.	Average	St.dev.	Average	St.dev.
A	0 (positive control)	0.5630	0.1240	0.4085	0.0283	0.4588	0.0723	0.3427	0.0283	0.2656	0.0046
B	10	0.0637	0.0058	0.3468	0.0333	0.4312	0.1101	0.3883	0.0295	0.5057	0.0481
C	20	0.5015	0.1396	0.3762	0.0644	0.4536	0.0940	0.4105	0.1153	0.4190	0.1163
D	40	0.3730	0.0381	0.4757	0.0052	0.4501	0.0664	0.4653	0.1536	0.3961	0.0412
E	80	0.0501	0.0017	0.2463	0.0143	0.3899	0.1082	0.5128	0.1344	0.0386	0.0026
F	160	0.0535	0.0057	0.0557	0.0057	0.3610	0.0144	0.4577	0.0480	0.0518	0.0019
G	320	0.5693	0.0606	0.0697	0.0039	0.1445	0.0047	0.0532	0.0042	0.0801	0.0049

Table S7. Raw absorbance data for *Arthrobacter oxydans* 3562 at 630 nm for DTA, DTThr, DTTyr4, DTTyr13 and DT4F, where row A represents the positive control (i.e. only strain), as well as 10% EtOH/H2O control and a blank control, i.e. where there is no bacterial strain.

Arthrobacter oxydans 3562

0h		DTA			DTThr			DTTyr4			DTTyr13			DT4F		
	**Concentration [μg/mL]**	*1*	*2*	*3*	*1*	*2*	*3*	*1*	*2*	*3*	*1*	*2*	*3*	*1*	*2*	*3*
**A**	0 (positive control)	*0.0673*	*0.0481*	*0.0618*	*0.1119*	*0.0968*	*0.0997*	*0.1052*	*0.1045*	*0.1009*	*0.0689*	*0.0818*	*0.0894*	*0.086*	*0.1051*	*0.0766*
**B**	10	*0.0643*	*0.0557*	*0.0602*	*0.1184*	*0.1092*	*0.1077*	*0.1167*	*0.1325*	*0.1194*	*0.0828*	*0.079*	*0.0894*	*0.0993*	*0.0985*	*0.0719*
**C**	20	*0.0809*	*0.0874*	*0.0711*	*0.1169*	*0.118*	*0.1026*	*0.1085*	*0.1309*	*0.1245*	*0.1*	*0.0914*	*0.0958*	*0.1002*	*0.1081*	*0.0916*
**D**	40	*0.0809*	*0.0646*	*0.0709*	*0.1129*	*0.1175*	*0.1034*	*0.1164*	*0.1186*	*0.1175*	*0.0981*	*0.0856*	*0.0922*	*0.1138*	*0.1067*	*0.1026*
**E**	80	*0.0745*	*0.0834*	*0.0658*	*0.1253*	*0.1277*	*0.1282*	*0.1362*	*0.1351*	*0.134*	*0.1206*	*0.1239*	*0.1264*	*0.1221*	*0.1341*	*0.1175*
**F**	160	*0.2558*	*0.0715*	*0.0951*	*0.1454*	*0.1409*	*0.1348*	*0.1696*	*0.1535*	*0.1545*	*0.1258*	*0.1307*	*0.1305*	*0.1316*	*0.1472*	*0.1392*
**G**	320	*0.2302*	*0.1642*	*0.1325*	*0.1842*	*0.1842*	*0.1766*	*0.2*	*0.2067*	*0.2206*	*0.1582*	*0.1685*	*0.1777*	*0.166*	*0.1821*	*0.1649*

24h		DTA			DTThr			DTTyr4			DTTyr13			DT4F		
	**Concentration [μg/mL]**	*1*	*2*	*3*	*1*	*2*	*3*	*1*	*2*	*3*	*1*	*2*	*3*	*1*	*2*	*3*
**A**	0 (positive control)	*0.1857*	*0.3031*	*0.1949*	*0.5465*	*0.5304*	*0.5799*	*0.5187*	*0.6152*	*0.4904*	*0.5146*	*0.4567*	*0.5444*	*0.5124*	*0.6251*	*0.6086*
**B**	10	*0.371*	*0.1047*	*0.1149*	*0.5016*	*0.5005*	*0.4655*	*0.5211*	*0.4943*	*0.4747*	*0.5145*	*0.4369*	*0.4995*	*0.4774*	*0.4663*	*0.5085*
**C**	20	*0.1281*	*0.128*	*0.112*	*0.4929*	*0.5197*	*0.4763*	*0.4862*	*0.4841*	*0.4818*	*0.5316*	*0.4884*	*0.4601*	*0.4709*	*0.4501*	*0.5283*
**D**	40	*0.1688*	*0.1067*	*0.1757*	*0.5019*	*0.4539*	*0.4921*	*0.4844*	*0.4285*	*0.4634*	*0.5683*	*0.4807*	*0.479*	*0.4436*	*0.4097*	*0.5199*
**E**	80	*0.0624*	*0.2713*	*0.0961*	*0.4553*	*0.4943*	*0.4697*	*0.4448*	*0.411*	*0.5008*	*0.606*	*0.5519*	*0.5631*	*0.4598*	*0.2941*	*0.2971*
**F**	160	*0.336*	*0.0507*	*0.0763*	*0.4277*	*0.4343*	*0.4293*	*0.451*	*0.4147*	*0.4246*	*0.5364*	*0.4245*	*0.4047*	*0.1692*	*0.1816*	*0.1794*
**G**	320	*0.2343*	*0.1303*	*0.0998*	*0.1853*	*0.1941*	*0.363*	*0.2232*	*0.2808*	*0.2301*	*0.1505*	*0.1672*	*0.1803*	*0.1874*	*0.214*	*0.1958*

0h		Control 10% EtOH/H_2O					Control blank				
	**Concentration [μg/mL]**	*1*	2	*3*	**Average**	**St.dev.**	**DTA**	**DTThr**	**DTTyr4**	**DTTyr13**	**DT4F**
**A**	0 (positive control)	*0.0687*	*0.0636*	*0.0618*	0.0647	0.0036	0.0283	0.0094	0.0107	0.0109	0.0127
**B**	10	*0.067*	*0.0757*	*0.0563*	0.0663	0.0097	0.0228	0.0158	0.0221	0.0213	0.017
**C**	20	*0.0703*	*0.0713*	*0.0673*	0.0696	0.0021	0.0326	0.0243	0.029	0.032	0.0297
**D**	40	*0.0707*	*0.0619*	*0.0642*	0.0656	0.0046	0.0177	0.0293	0.0355	0.0369	0.0368
**E**	80	*0.08*	*0.069*	*0.0711*	0.0734	0.0058	0.0288	0.0523	0.0628	0.0687	0.0601
**F**	160	*0.0734*	*0.0635*	*0.0724*	0.0698	0.0055	0.0337	0.0679	0.0787	0.0726	0.0799
**G**	320	*0.0757*	*0.0809*	*0.0766*	0.0777	0.0028	0.0775	0.1017	0.1394	0.1244	0.1198

24h		Control 10% EtOH/H_2O					Control blank				
	**Concentration [µg/mL]**	***1***	**2**	***3***	**Average**	**St.dev.**	**DTA**	**DTThr**	**DTTyr4**	**DTTyr13**	**DT4F**
**A**	0 (positive control)	*0.1065*	*0.1631*	*0.1048*	0.1248	0.0332	0.6065	0.177	0.022	0.1544	0.3579
**B**	10	*0.1838*	*0.1571*	*0.1288*	0.1566	0.0275	0.634	0.2063	0.1582	0.3092	0.0403
**C**	20	*0.1407*	*0.1602*	*0.1362*	0.1457	0.0128	0.5954	0.0327	0.1271	0.2608	0.2993
**D**	40	*0.1365*	*0.1226*	*0.1128*	0.1240	0.0119	0.218	0.0384	0.0936	0.2831	0.2462
**E**	80	*0.1539*	*0.1391*	*0.176*	0.1563	0.0186	0.4213	0.0572	0.0721	0.2101	0.2036
**F**	160	*0.1672*	*0.1243*	*0.2633*	0.1849	0.0712	0.0759	0.122	0.0792	0.0701	0.0863
**G**	320	*0.1686*	*0.3691*	*0.1255*	0.2211	0.1300	0.0715	0.0773	0.1168	0.1176	0.0956

Table S8. Average values with standard deviation of peptides' absorbance for *Arthrobacter oxydans* 3562 at 630 nm.

0h		DTA		DTThr		DTTyr4		DTTyr13		DT4F	
	**Concentration [µg/mL]**	**Average**	**St.dev.**	**Average**	**St.dev.**	**Average**	**St.dev.**	**Average**	**St.dev.**	**Average**	**St.dev.**
**A**	0 (positive control)	0.0506	0.0083	0.0422	0.0020	0.0393	0.0037	0.0385	0.0019	0.0425	0.0023
**B**	10	0.0503	0.0080	0.0416	0.0014	0.0411	0.0028	0.0408	0.0012	0.0434	0.0019
**C**	20	0.0514	0.0023	0.0440	0.0074	0.0470	0.0016	0.0402	0.0021	0.0454	0.0030
**D**	40	0.0509	0.0036	0.0437	0.0026	0.0526	0.0024	0.0407	0.0016	0.0443	0.0012
**E**	80	0.0633	0.0079	0.0519	0.0040	0.0685	0.0013	0.0505	0.0015	0.0592	0.0012
**F**	160	0.0818	0.0152	0.0638	0.0035	0.0944	0.0122	0.0564	0.0017	0.0737	0.0030
**G**	320	0.0971	0.0085	0.0941	0.0067	0.1681	0.0031	0.0736	0.0025	0.1138	0.0056

24h		DTA		DTThr		DTTyr4		DTTyr13		DT4F	
	**Concentration [µg/mL]**	**Average**	**St.dev.**	**Average**	**St.dev.**	**Average**	**St.dev.**	**Average**	**St.dev.**	**Average**	**St.dev.**
A	0 (positive control)	0.2279	0.0653	0.5523	0.0252	0.5414	0.0654	0.5052	0.0446	0.5820	0.0609
B	10	0.1969	0.1509	0.4892	0.0205	0.4967	0.0233	0.4836	0.0412	0.4841	0.0219
C	20	0.1227	0.0093	0.4963	0.0219	0.4840	0.0022	0.4934	0.0360	0.4831	0.0405
D	40	0.1504	0.0380	0.4826	0.0254	0.4588	0.0282	0.5093	0.0511	0.4577	0.0564
E	80	0.1433	0.1122	0.4731	0.0197	0.4522	0.0454	0.5737	0.0286	0.3503	0.0948
F	160	0.1543	0.1578	0.4304	0.0034	0.4301	0.0188	0.4552	0.0710	0.1767	0.0066
G	320	0.1548	0.0705	0.2475	0.1002	0.2447	0.0315	0.1660	0.0149	0.1991	0.0136

Table S9. Raw absorbance data for *Candida albicans* 74 at 630 nm for DTA, DTThr, DTTyr4, DTTyr13 and DT4F, where row A represents the positive control (i.e. only strain), as well as 10% EtOH/H2O control and a blank control, i.e. where there is no bacterial strain.

Arthrobacter oxydans																
**0h**		**DTA**			**DTThr**			**DTTyr4**			**DTTyr13**			**DT4F**		
	**Concentration [μg/mL]**	***1***	***2***	***3***	***1***	***2***	***3***	***1***	***2***	***3***	***1***	***2***	***3***	***1***	***2***	***3***
**A**	0 (positive control)	*0.0542*	*0.0578*	*0.059*	*0.0833*	*0.1038*	*0.0896*	*0.0894*	*0.0827*	*0.0784*	*0.0843*	*0.0737*	*0.0833*	*0.1134*	*0.0922*	*0.0984*
**B**	10	*0.0643*	*0.0562*	*0.0619*	*0.0905*	*0.1004*	*0.0864*	*0.0968*	*0.0793*	*0.0921*	*0.0915*	*0.0757*	*0.0783*	*0.0863*	*0.0885*	*0.1048*
**C**	20	*0.0658*	*0.064*	*0.0614*	*0.0855*	*0.0468*	*0.101*	*0.1042*	*0.0843*	*0.0892*	*0.0807*	*0.0805*	*0.0812*	*0.1025*	*0.0947*	*0.1066*
**D**	40	*0.0713*	*0.068*	*0.0654*	*0.0885*	*0.1007*	*0.104*	*0.1099*	*0.0943*	*0.0875*	*0.0919*	*0.0787*	*0.0801*	*0.0869*	*0.1044*	*0.1053*
**E**	80	*0.0893*	*0.0813*	*0.0778*	*0.0897*	*0.0883*	*0.0806*	*0.1038*	*0.1107*	*0.1108*	*0.1053*	*0.0909*	*0.0957*	*0.1001*	*0.0982*	*0.1185*
**F**	160	*0.0919*	*0.099*	*0.0901*	*0.0945*	*0.0903*	*0.1046*	*0.1239*	*0.1491*	*0.1606*	*0.0939*	*0.0969*	*0.0828*	*0.0975*	*0.1133*	*0.1121*
**G**	320	*0.1425*	*0.1207*	*0.111*	*0.1351*	*0.133*	*0.1247*	*0.2095*	*0.2197*	*0.2119*	*0.0943*	*0.0888*	*0.0945*	*0.0922*	*0.096*	*0.1105*

**24h**		**DTA**			**DTThr**			**DTTyr4**			**DTTyr13**			**DT4F**		
	**Concentration [μg/mL]**	***1***	***2***	***3***	***1***	***2***	***3***	***1***	***2***	***3***	***1***	***2***	***3***	***1***	***2***	***3***
**A**	0 (positive control)	*1.1823*	*1.0872*	*1.0953*	*1.3711*	*1.3965*	*1.3245*	*1.3612*	*1.3708*	*1.2979*	*1.3938*	*1.3514*	*1.353*	*1.3905*	*1.2909*	*1.2965*
**B**	10	*1.2004*	*1.1671*	*1.1428*	*1.3352*	*1.324*	*1.3202*	*1.3032*	*1.3303*	*1.333*	*1.3028*	*1.3632*	*1.2711*	*1.3824*	*1.3398*	*1.318*
**C**	20	*1.1797*	*1.0774*	*1.0837*	*1.3442*	*0.0597*	*1.3652*	*1.3035*	*1.3372*	*1.3724*	*1.3583*	*1.3525*	*1.3394*	*1.4402*	*1.3971*	*1.3645*
**D**	40	*1.157*	*1.0849*	*1.1192*	*1.3272*	*1.3206*	*1.3291*	*1.321*	*1.3344*	*1.3324*	*1.3384*	*1.3011*	*1.3108*	*1.3779*	*1.3611*	*1.3514*
**E**	80	*1.1204*	*1.1085*	*1.1017*	*1.3359*	*1.3181*	*1.2984*	*1.3154*	*1.3003*	*1.3111*	*1.4045*	*1.4009*	*1.403*	*1.3867*	*1.3425*	*1.3973*
**F**	160	*1.0912*	*1.0646*	*1.0902*	*1.1911*	*1.1884*	*1.2452*	*1.2692*	*1.2546*	*1.2433*	*1.3627*	*1.3908*	*1.3498*	*1.196*	*1.288*	*1.274*
**G**	320	*0.5691*	*0.6081*	*0.5523*	*1.1878*	*1.1912*	*1.181*	*1.229*	*1.2336*	*1.2053*	*1.3281*	*1.2894*	*1.3206*	*1.256*	*1.3196*	*1.3363*

**0h**		**Control 10% EtOH/H_2O**					**Control blank**				
	**Concentration [μg/mL]**	***1***	***2***	***3***	**Average**	**St.dev.**	**DTA**	**DTThr**	**DTTyr4**	**DTTyr13**	**DT4F**
**A**	0 (positive control)	*0.0751*	*0.0757*	*0.0749*	0.0752	0.0004	0.0337	0.0339	0.0322	0.0335	0.0448
**B**	10	*0.075*	*0.0665*	*0.0716*	0.0710	0.0043	0.0354	0.0374	0.0324	0.0308	0.0506
**C**	20	*0.0765*	*0.0806*	*0.0904*	0.0825	0.0071	0.0477	0.04	0.046	0.0402	0.0573
**D**	40	*0.0706*	*0.0702*	*0.0602*	0.0670	0.0059	0.0499	0.0361	0.0428	0.0403	0.0382
**E**	80	*0.0786*	*0.071*	*0.0623*	0.0706	0.0082	0.0566	0.0547	0.0611	0.0429	0.0522
**F**	160	*0.0926*	*0.083*	*0.0665*	0.0807	0.0132	0.0594	0.059	0.0737	0.0373	0.062
**G**	320	*0.0837*	*0.0765*	*0.0728*	0.0777	0.0055	0.0982	0.0828	0.1001	0.0443	0.0701

24h		Control 10% EtOH/H_2O					Control blank				
	**Concentration [µg/mL]**	*1*	*2*	*3*	**Average**	**St.dev.**	**DTA**	**DTThr**	**DTTyr4**	**DTTyr13**	**DT4F**
**A**	0 (positive control)	*1.1295*	*1.2345*	*1.1868*	1.1836	0.0526	0.0702	0.994	0.266	0.1542	0.1596
**B**	10	*1.1129*	*1.1419*	*1.1307*	1.1285	0.0146	0.857	0.3812	0.257	0.2283	0.0815
**C**	20	*1.1096*	*1.0843*	*1.158*	1.1173	0.0374	0.9422	0.7988	0.0783	0.7345	0.1839
**D**	40	*1.1269*	*1.1246*	*1.1364*	1.1293	0.0063	0.0289	0.4529	0.683	0.2408	0.2518
**E**	80	*1.2267*	*1.1134*	*1.165*	1.1684	0.0567	0.0417	0.2255	0.1295	0.0503	0.0641
**F**	160	*1.1262*	*1.164*	*1.1634*	1.1512	0.0217	0.0459	0.1471	0.0898	0.1332	0.0754
**G**	320	*1.1852*	*1.1522*	*1.1819*	1.1731	0.0182	0.0779	0.0926	0.1093	0.0576	0.0824

Table S10. Average values with standard deviation of peptides' absorbance for *Candida albicans* 74at 630 nm.

0h		DTA		DTThr		DTTyr4		DTTyr13		DT4F	
	**Concentration [µg/mL]**	**Average**	**St.dev.**	**Average**	**St.dev.**	**Average**	**St.dev.**	**Average**	**St.dev.**	**Average**	**St.dev.**
**A**	0 (positive control)	0.0570	0.0025	0.0922	0.0105	0.0835	0.0055	0.0804	0.0059	0.1013	0.0109
**B**	10	0.0608	0.0042	0.0924	0.0072	0.0894	0.0091	0.0818	0.0085	0.0932	0.0101
**C**	20	0.0637	0.0022	0.0778	0.0279	0.0926	0.0104	0.0808	0.0004	0.1013	0.0060
**D**	40	0.0682	0.0030	0.0977	0.0082	0.0972	0.0115	0.0836	0.0073	0.0989	0.0104
**E**	80	0.0828	0.0059	0.0862	0.0049	0.1084	0.0040	0.0973	0.0073	0.1056	0.0112
**F**	160	0.0937	0.0047	0.0965	0.0074	0.1445	0.0188	0.0912	0.0074	0.1076	0.0088
**G**	320	0.1247	0.0161	0.1309	0.0055	0.2137	0.0053	0.0925	0.0032	0.0996	0.0097

24h		DTA		DTThr		DTTyr4		DTTyr13		DT4F	
	**Concentration [µg/mL]**	**Average**	**St.dev.**	**Average**	**St.dev.**	**Average**	**St.dev.**	**Average**	**St.dev.**	**Average**	**St.dev.**
**A**	0 (positive control)	1.1216	0.0527	1.3640	0.0365	1.3433	0.0396	1.3661	0.0240	1.3260	0.0560
**B**	10	1.1701	0.0289	1.3265	0.0078	1.3222	0.0165	1.3124	0.0468	1.3467	0.0328
**C**	20	1.1136	0.0573	0.9230	0.7477	1.3377	0.0345	1.3501	0.0097	1.4006	0.0380
**D**	40	1.1204	0.0361	1.3256	0.0045	1.3293	0.0072	1.3168	0.0194	1.3635	0.0134
**E**	80	1.1102	0.0095	1.3175	0.0188	1.3089	0.0078	1.4028	0.0018	1.3755	0.0291
**F**	160	1.0820	0.0151	1.2082	0.0320	1.2557	0.0130	1.3678	0.0210	1.2527	0.0496
**G**	320	0.5765	0.0286	1.1867	0.0052	1.2226	0.0152	1.3127	0.0205	1.3040	0.0424
